# Genome-wide analysis of *Mycobacterium tuberculosis* polymorphisms reveals lineage-specific associations with drug resistance

**DOI:** 10.1186/s12864-019-5615-3

**Published:** 2019-03-29

**Authors:** Yaa E. A. Oppong, Jody Phelan, João Perdigão, Diana Machado, Anabela Miranda, Isabel Portugal, Miguel Viveiros, Taane G. Clark, Martin L. Hibberd

**Affiliations:** 1 Pathogen Molecular Biology Department, Faculty of Infectious and Tropical Diseases, London School of Hygiene and Tropical Medicine, Keppel Street, London, WC1E 7HT UK; 2 iMed.ULisboa – Research Institute for Medicines, Faculdade de Farmácia, Universidade de Lisboa, Lisbon, Portugal; 3 Global Health and Tropical Medicine, Instituto de Higiene e Medicina Tropical, Universidade Nova de Lisboa, UNL, Lisbon, Portugal; 4 National Mycobacterium Reference Laboratory, Porto, Portugal; 5 Faculty of Epidemiology and Population Health, LSHTM, London, UK

**Keywords:** Drug resistance, Evolution, Mutations, *Mycobacterium tuberculosis*, Tuberculosis

## Abstract

**Background:**

Continuing evolution of *the Mycobacterium tuberculosis (Mtb)* complex genomes associated with resistance to anti-tuberculosis drugs is threatening tuberculosis disease control efforts. Both multi- and extensively drug resistant *Mtb* (MDR and XDR, respectively) are increasing in prevalence, but the full set of *Mtb* genes involved are not known. There is a need for increased sensitivity of genome-wide approaches in order to elucidate the genetic basis of anti-microbial drug resistance and gain a more detailed understanding of *Mtb* genome evolution in a context of widespread antimicrobial therapy. Population structure within the *Mtb* complex, due to clonal expansion, lack of lateral gene transfer and low levels of recombination between lineages, may be reducing statistical power to detect drug resistance associated variants.

**Results:**

To investigate the effect of lineage-specific effects on the identification of drug resistance associations, we applied genome-wide association study (GWAS) and convergence-based (PhyC) methods to multiple drug resistance phenotypes of a global dataset of *Mtb* lineages 2 and 4*,* using both lineage-wise and combined approaches. We identify both well-established drug resistance variants and novel associations; uniquely identifying associations for both lineage-specific and -combined GWAS analyses. We report 17 potential novel associations between antimicrobial resistance phenotypes and *Mtb* genomic variants.

**Conclusions:**

For GWAS, both lineage-specific and -combined analyses are useful, whereas PhyC may perform better in contexts of greater diversity. Unique associations with XDR in lineage-specific analyses provide evidence of diverging evolutionary trajectories between lineages 2 and 4 in response to antimicrobial drug therapy.

**Electronic supplementary material:**

The online version of this article (10.1186/s12864-019-5615-3) contains supplementary material, which is available to authorized users.

## Background

Despite clonal expansion and a lack of lateral gene transfer in *Mycobacterium tuberculosis* (*Mtb),* the evolution of drug resistance is threatening tuberculosis disease (TB) control efforts. Resistance to all anti-*Mtb* drugs has been observed, usually evolving relatively shortly after their introduction. Drug-resistant TB is phenotypically categorised as multi-drug resistant (MDR) when resistant to two first–line drugs, rifampicin and isoniazid; extensively drug-resistant (XDR) occurs when MDR *Mtb* have additional resistance to fluoroquinolones and at least one second-line injectable. Only 50% of patients receiving treatment for MDR TB, globally, were successfully treated in 2014 [1].

De novo emergence of drug resistance has been observed, with the presence of multiple unfixed drug-resistance mutations and selective sweeps in *Mtb* populations within patients [2–4]. Additionally, transmission of resistant strains is frequently observed [5, 6]. Indeed, many mutations associated with antimicrobial resistance have been identified [7], some have been associated with no fitness cost and others with additional compensatory mutations that may increase fitness and enable transmission [8]. These polymorphisms include both point mutations, for example, single nucleotide polymorphisms (SNPs) such as in *rpoB* [9] and structural variants such as the *dfrA-thyA* double deletion linked to para-aminosalicylic acid resistance [10]. Genes involved in resistance to some drugs are well known; for example, mutations for rifampicin (in *rpoB* and *rpoC*) and isoniazid (in *katG*) are well characterised [7]. However, the mechanisms for ethambutol (*embB*), pyrazinamide (*pncA*) and second line drug resistance are not fully known. As whole genome sequencing of *Mtb* becomes more routinely applied [11], association approaches using genomic variation have the potential to provide new insights into these resistance mechanisms. Compensatory mutations such as those in *rpoA* and *rpoC,* associated with the *rpoB* rifampicin resistance mutations, have been associated with transmission of drug resistant strains [12]. Furthermore, as patients receive a cocktail of anti-*Mtb* drugs, multiple concomitant resistance can arise naturally, and this complicates the analysis of phenotype-genotype relationships [13].

The genome-wide association study (GWAS) approach has been widely used in human genetics; for example, to identify variants in the class II human leukocyte antigens (HLA) region associated with susceptibility to TB infection [14]. However, it is increasingly being applied to pathogen research and shows great promise [13, 15, 16]. It allows the identification of variants across the genome, associated with specific phenotypes. In order to prevent spurious associations, pathogen GWASs face the need to deal with the much higher levels of population structure seen in bacteria compared to humans, whilst maximising sensitivity [17, 18]. This is especially important for *Mtb* due to its clonality. This clonality is consistent with a phylogenetic tree structure and thus has led to the application of convergence-based methods, which have identified resistance mutations in *Mtb* [13, 19]. Such methods seek to identify convergent evolution in genetically diverse strains with similar resistance phenotypes. This happens when mutations in the same gene or nucleotide position occur repeatedly and independently become fixed, thus signaling their positive selection for a particular phenotype.

However, there remain questions as to the importance of historic genetic background variation in the evolution of drug resistance, such as between *Mtb* lineages, which have not been systematically explored [20]. The *Mtb* complex is categorised into seven lineages, defined on the basis of molecular typing, which are endemic in different locations around the globe. These lineages are known to have other distinctive features, with some persisting in geographical regions (lineages 5 and 6 in West Africa) and others spreading across continents (lineage 2- East Asian and lineage 4 – Euro-American strains). This observation has led to the hypothesis that the strain-types are specifically adapted to people of different genetic backgrounds [21]. These lineages may vary in their propensity to transmit, their virulence, site of infection and ultimately propensity to cause disease [22–24], but results are inconsistent and there is considerable inter-strain variation within lineages [25, 26]. Recent research into lineage 4 alludes to this variation, suggesting different evolutionary strategies are employed by different sublineages [27]. A set of single nucleotide polymorphisms (SNPs) has been identified that can be used to barcode sub-lineages [28], leading to informatic tools that position sequenced samples within a global phylogeny [29]. Thus, lineage-based genetic differences may also be important in resistance adaptations to anti-*Mtb* drug exposure.

The current study applies lineage-specific and lineage-combined GWAS, alongside convergence-based PhyC methods, to gain insight into lineage-specific drug resistance evolution. We focus on the modern lineage 2 and lineage 4 isolates, which are known to be drug resistant globally, and use a large dataset involving *Mtb* isolate sequences from more than 12 countries (*n* > 4400).

## Results

### Genomic variants and population structure

**Fig. 1:**
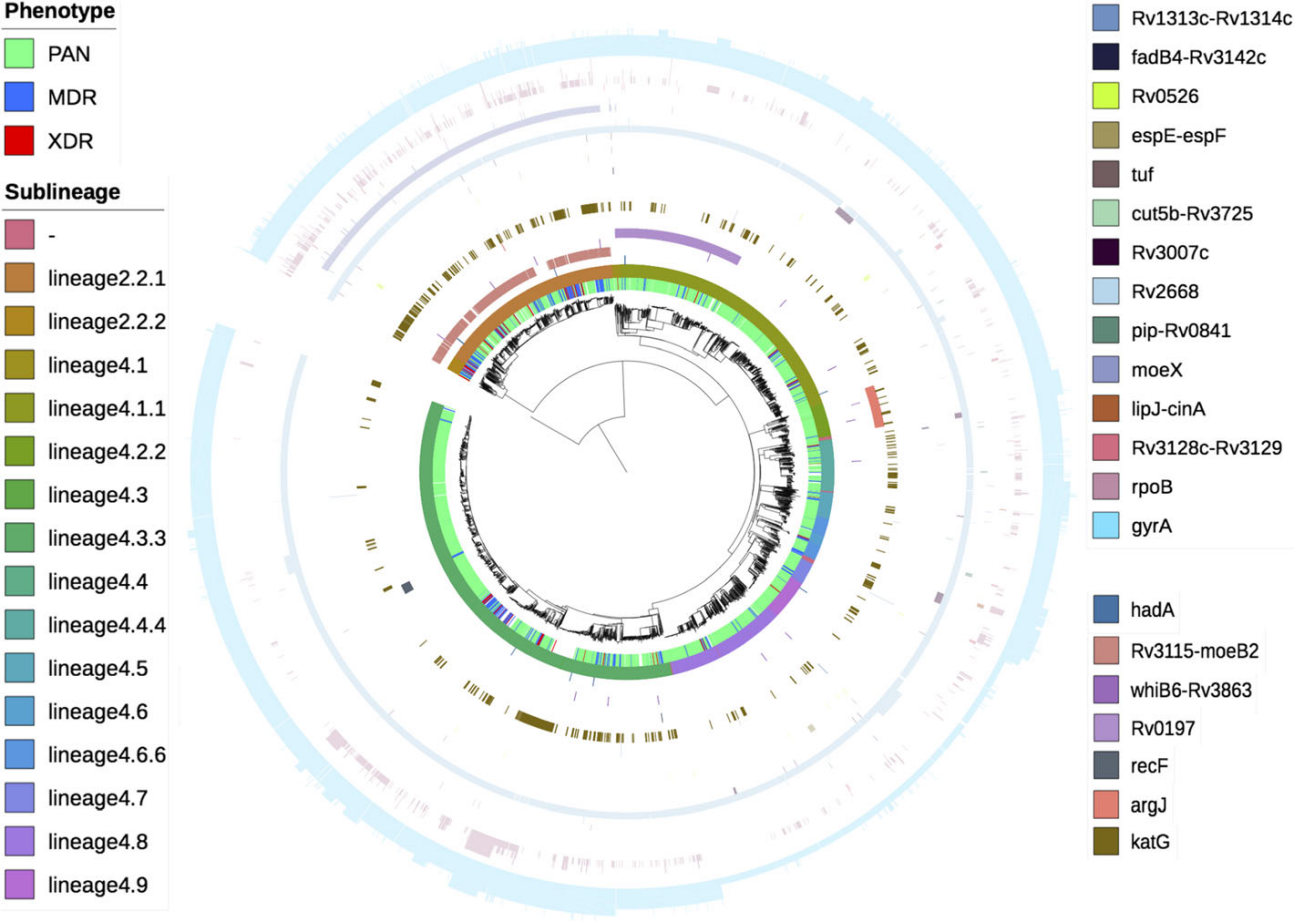
Phylogenetic tree of all samples: coloured by phenotype, sublineage and genotype for novel variants alongside *katG*, *rpoB* and *gyrA*. From inner to outer, each track is coloured by; drug-resistance phenotype, sublineage, variant genotype for; *hadA* (732110), Rv3115-moeB2 (3482717), *whiB6-Rv3863* (4338594), *Rv0197* (232574), *recF* (4047), *argJ* (1867614), *katG* (2155168); locus genotype for; *Rv1313c-Rv1314c*, *fadB4-Rv3142c*, *Rv0526*, *espE-espF*, *tuf*, *cut5b-Rv3725*, *Rv3007c*, *Rv2668*, *pip-Rv0841*, *moeX*, *lipJ-cinA*, *Rv3128c-Rv3129*, *rpoB*, *gyrA*. Variant genotype is coloured in samples where a non-reference variant is present with respect to H37rv reference and variant position is given in brackets. Locus genotype is coloured in samples with one or more non-synonymous or intergenic variants at each locus with respect to H37rv reference, relative height of the bar reflects number of variants at each loci

High quality SNP and insertion and deletion (indel) variants were characterised in relation to the H37Rv reference genome, from raw sequence data from a convenience sample of existing data for isolates in lineages 2 (*n* = 702) and 4 (*n* = 3706). These isolates are within a global drug resistance data set [13], which has been further complemented by additional phenotypic data (see Methods). After removing variants that are monomorphic within each dataset, the final lineage-combined dataset consisted of 157,726 SNPs, 5998 deletions and 2926 insertions across the 4408 isolates (see Additional file 1). The median number of SNPs per sample in the lineage 2 dataset, after removing monomorphic variants, was 332 (range: 189–386) and in lineage 4 was 724 (range: 10–870) (significant difference between lineages with Wilcoxon test *p*-value < minimum calculable (2.2 × 10^− 16^)). Lineage 4 contains the H37Rv reference strain, but also has increased strain-type diversity [13, 28]. The median number of indels per sample in lineage 2 was 31 (range: 7–42) and in lineage 4 was 40 (range: 2–61) (significant difference between lineages Wilcoxon test: p-value < minimum calculable (2.2 × 10^− 16^)) (see Additional file 1). The majority of variants were rare, with 75% of them found to have a non-reference variant frequency (defined as the number of isolates with a non-reference allele at a specific variant position divided by the total number of isolates with a non-missing allele at this position) of less than 0.0028 and 0.00054 in lineages 2 and 4, respectively (see Additional file 1 and Additional file 2). A principal component analysis (PCA) using the variants revealed the expected clustering by lineage and greater diversity within lineage 4 (see Additional file 3). Within lineage 2, the first 10 principal components account for 71.9% of the variation (see Additional file 3 and Additional file 4) and the mean pairwise variant distance was 1074 (range: 0–6270) (see Additional file 3). Within lineage 4, the first 10 principal components account for 88.9% of the variation (see Additional file 3 and Additional file 4) and the mean pairwise variant distance was 1458 (range: 0–11,780) (see Additional file 3**)**. There are 567 isolates with < 10 variants different from at least one other isolate, indicative of potential transmission events, which can confound an association analysis. A phylogenetic tree constructed using the variants mimicked the relationships observed in the PCA, with isolates clustering by sublineage on both (see Additional file 3 and Fig. 1).

### Drug resistance phenotypes

Overall, analyses were conducted for 17 drug resistance phenotypes, including for 12 individual drugs and 5 composite phenotypes. The 12 individual drug resistance phenotypes with frequency of resistance ranging from 3.3% (MOX in lineage 4) to 43.0% (STM in lineage 2), and the composite phenotypes of MDR (lineage 2 35.7%; lineage 4 9.5%) and XDR (lineage 2 9.9%; lineage 4 1.2%). The combined second-line drug resistance phenotypes for resistance to any fluoroquinones (FQ) and resistance to any aminoglycosides (AG) were also considered (see Additional file 5). The completeness of drug-resistance phenotype data is variable. Rifampicin was the most tested for (tested for in 92.0% of isolates); while ciprofloxacin was the least (tested for in 4.2% of isolates) (see Additional file 6). Furthermore, there is evidence of multiple concomitant resistance with 44.1% of MDR isolates also resistant to ethambutol.

### Convergence-based analyses, variant-based GWAS and locus-based identified known resistance conferring variants

We performed convergence-based analyses (PhyC), GWAS across loci (locus-based) and GWAS on individual variants (variant-based). Each were conducted in a lineage-specific and lineage-combined manner. Due to the close relatedness between some samples, for the GWAS analyses, we applied specialized regression models with random effects that have been implemented in a human setting to handle “cryptic relatedness” [13] (see Methods).

In total, PhyC analysis of the combined lineages identified 53 variants in 20 different loci, with individual lineage analyses identifying a subset of these loci (see Table 1**,** Additional file 7). Eleven of these loci were not identified by GWAS techniques, including eight loci with known involvement in antimicrobial resistance; *thyX-hsdS.1* (para-aminosalicylic acid)*, rpoC* (rifampicin)*, pncA-Rv2044c* (pyrazinamide)*, eis-Rv2417c* (aminoglycosides)*, folC* (para-aminosalicylic acid)*, fabG1* (isoniazid)*, oxyR’-ahpC* (isoniazid) and *gyrB* (fluoroquinolones) (see Table 1**,** Additional file 8).

**Table 1:** Significant associations between genomic variants and drug resistance phenotypes identified by PhyC

Locus	Known Phenotype Association [13]	Lineage	Observed Phenotype Association (position; *p*-value)	Total
*rpoB*	RMP	2	INH(761,155;1.2 × 10^− 06^, 761,139;5.6 × 10^− 06^), MDR(761,155;1.5 × 10^− 12^, 761,139;1.4 × 10^− 07^, 761,140;5.5 × 10^− 04^), RMP(761,155;2.4 × 10^− 11^, 761,139;6.7 × 10^− 09^, 761,110;4.6 × 10^− 04^, 761,140;9.3 × 10^− 04^), XDR(761,155;2.6 × 10^− 04^, 761,139;7.5 × 10^− 04^)	11
*rpoB*	RMP	4	AG(761,155;2.0 × 10^−5^), EMB(761,155;2.2 × 10^− 14^), INH(761,155;3.4 × 10^− 32^, 761,139;6.2 × 10^− 11^, 761,110;7.6 × 10^− 08^, 761,140;3.3 × 10^− 07^), MDR(761,155;2.1 × 10^− 45^, 761,139;2.8 × 10^− 20^, 761,140;1.3 × 10^− 09^, 761,110;1 × 10^− 08^, 759,939;2.8 × 10^− 04^), PZA(761,155;1.5 × 10^− 12^, 761,110;2.2 × 10^− 06^), RMP(761,155;5.7 × 10^− 54^, 761,139;9.7 × 10^− 27^, 761,110;2.6 × 10^− 12^, 761,140;3.4 × 10^− 11^, 761,998;1.1 × 10^− 05^, 761,109;7.3 × 10^− 05^, 759,939;4.9 × 10^− 04^), STRx10P(761,155;2.1 × 10^− 16^, 761,110;7.5 × 10^− 04^), XDR(761,155;7.1 × 10^− 14^, 761,110;2.7 × 10^− 05^)	23
*rpoB*	RMP	2 + 4	AG(761,155;2.3 × 10^−5^), EMB(761,155;3.8 × 10^− 18^, 761,140;6.3 × 10^− 06^, 761,110;1.1 × 10^− 05^, 761,139;2 × 10^− 05^), INH(761,155;2.3 × 10^− 42^, 761,139;3.5 × 10^− 19^, 761,140;2.3 × 10^− 11^, 761,110;1.1 × 10^− 09^, 761,161;1.5 × 10^− 06^), MDR(761,155;7.8 × 10^− 63^, 761,139;1.7 × 10^− 31^, 761,140;4.8 × 10^− 15^, 761,110;1.3 × 10^− 13^, 761,161;5.8 × 10^− 05^, 761,095;1.8 × 10^− 04^, 759,939;2.6 × 10^− 04^, 761,109;2.6 × 10^− 04^), PZA(761,155;5.6 × 10^− 16^, 761,110;2 × 10^− 07^, 761,139;5.8 × 10^− 04^), RMP(761,155;3.5 × 10^− 70^, 761,139;3.2 × 10^− 38^, 761,110;2.4 × 10^− 17^, 761,140;2.2 × 10^− 15^, 761,161;4.4 × 10^− 07^, 761,109;3.5 × 10^− 05^, 761,998;1 × 10^− 04^, 761,095;3.1 × 10^− 04^, 759,939;4.7 × 10^− 04^, 760,314;4.7 × 10^− 04^), STM(761,155;8.4 × 10^− 21^, 761,110;2.1 × 10^− 07^, 761,139;2.6 × 10^− 07^, 761,140;8.5 × 10^− 05^, 761,161;1.8 × 10^− 04^), XDR(761,155;2.2 × 10^− 18^, 761,110;2.6 × 10^− 08^, 761,139;9.7 × 10^− 08^, 761,161;1.9 × 10^− 06^, 761,109;6.2 × 10^− 05^)	40
*embB*	EMB	2	EMB(4,247,429;3 × 10^− 07^, 4,247,431;1.8 × 10^− 04^), INH(4,247,429;9.3 × 10^− 10^, 4,247,431;3.1 × 10–05), MDR(4,247,429;2.5 × 10^− 08^, 4,247,431;1.1 × 10^− 04^), RMP(4,247,429;7.6 × 10^− 09^, 4,247,431;1.3 × 10^− 04^, 4,247,730;8.7 × 10^− 04^), STM(4,247,429;1 × 10^− 05^, 4,247,431;1.1 × 10^− 04^), XDR(4,247,429;4.3 × 10^− 06^, 4,247,431;1.2 × 10^− 04^, 4,247,730;1.2 × 10^− 04^)	14
*embB*	EMB	4	AG(4,247,431;7.1 × 10^− 4^), EMB(4,247,431;3 × 10^− 11^, 4,247,729;3 × 10^− 08^, 4,247,730;1 × 10^− 07^, 4,248,003;1 × 10^− 07^, 4,247,429;1.5 × 10^− 06^, 4,247,574;8.1 × 10^− 04^), INH(4,247,431;6.6 × 10^− 19^, 4,247,730;1.4 × 10^− 09^, 4,247,429;1.7 × 10^− 09^, 4,247,729;6.3 × 10^− 06^, 4,247,574;1.8 × 10^− 05^, 4,248,003;2.8 × 10^− 05^), MDR(4,247,431;2.3 × 10^− 21^, 4,247,429;1.5 × 10^− 09^, 4,247,730;8.8 × 10^− 08^, 4,247,574;6 × 10^− 07^, 4,247,729;4.2 × 10^− 06^, 4,248,003;6 × 10^− 04^), PZA(4,247,431;1.2 × 10^− 04^, 4,247,730;2.2 × 10^− 04^, 4,248,003;5.3 × 10^− 04^), RMP(4,247,431;2.2 × 10^− 18^, 4,247,429;1.5 × 10^− 10^, 4,247,730;1.4 × 10^− 08^, 4,247,574;6.6 × 10^− 05^, 4,247,729;1.3 × 10^− 04^, 4,248,003;1.5 × 10^− 04^), STM(4,247,431;1.5 × 10^− 08^, 4,247,729;3.6 × 10^− 06^, 4,247,574;7.2 × 10–^04^), XDR(4,247,429;1.3 × 10^− 04^)	31
*embB*	EMB	2 + 4	AG(4,247,431;3.5 × 10^−4^), EMB(4,247,429;9.2 × 10^−21^, 4,247,431;1.5 × 10^− 16^, 4,247,729;2.1 × 10^− 09^, 4,247,730;6.4 × 10^− 08^, 4,248,003;2.8 × 10^− 07^, 4,247,574;1.4 × 10^− 04^, 4,249,518;1.9 × 10^− 04^), FQ(4,247,730;9.5 × 10^− 07^), INH(4,247,429;2.7 × 10^− 27^, 4,247,431;1 × 10^− 25^, 4,247,730;8.4 × 10^− 14^, 4,248,003;1.2 × 10^− 08^, 4,247,729;1.4 × 10^− 07^, 4,247,574;1.7 × 10^− 07^), MDR(4,247,431;3.2 × 10^− 26^, 4,247,429;6.1 × 10^− 26^, 4,247,730;2 × 10^− 12^, 4,247,574;2.5 × 10^− 09^, 4,247,729;1.3 × 10^− 07^, 4,248,003;1.5 × 10^− 07^), PZA(4,247,730;6.3 × 10^− 08^, 4,247,431;2 × 10^− 05^, 4,247,429;2.9 × 10^− 04^, 4,248,003;4.6 × 10^− 04^), RMP(4,247,429;4.1 × 10^− 29^, 4,247,431;4.8 × 10^− 24^, 4,247,730;3.1 × 10^− 13^, 4,248,003;3.5 × 10^− 07^, 4,247,574;4.7 × 10^− 07^, 4,247,729;2.5 × 10^− 06^, 4,247,469;4.7 × 10^− 04^), STRx10P(4,247,431;2.2 × 10^− 14^, 4,247,429;2.9 × 10^− 13^, 4,247,729;1.4 × 10^− 05^, 4,248,003;2.6 × 10^− 05^, 4,247,730;5.5 × 10^− 05^, 4,247,574;6.9 × 10^− 05^), XDR(4,247,429;4.4 × 10^− 13^, 4,247,431;8.9 × 10^− 10^, 4,247,730;2.6 × 10^− 08^)	41
*katG*	INH	2	INH(2,155,168;2.7 × 10^− 07^), MDR(2,155,168;4.5 × 10^− 08^), RMP(2,155,168;5.7 × 10^− 04^), STM(2,155,168;8.3 × 10^− 04^), XDR(2,155,168;4.1 × 10^− 09^)	5
*katG*	INH	4	EMB(2,155,168;1.5 × 10^− 07^), INH(2,155,168;2 × 10^− 63^, 2,155,167;8.5 × 10^− 05^), MDR(2,155,168;3 × 10^− 58^, 2,155,167;2.8 × 10^− 04^), PZA(2,155,168;1.5 × 10^− 09^), RMP(2,155,168;2.9 × 10^− 29^), STRx10P(2,155,168;2.8 × 10^− 11^), XDR(2,155,168;1.8 × 10^− 14^)	9
*katG*	INH	2 + 4	EMB(2,155,168;4.8 × 10^− 11^), INH(2,155,168;7.1 × 10^− 72^, 2,155,167;1.1 × 10^− 04^), MDR(2,155,168;3.3 × 10^− 68^, 2,155,167;2.6 × 10^− 04^), PZA(2,155,168;1.7 × 10^− 11^), RMP(2,155,168;2.5 × 10^− 36^), STRx10P(2,155,168;3.9 × 10^− 18^), XDR(2,155,168;3.5 × 10^− 28^)	9
*rpsL*	STM	2	INH(781,687;5.9 × 10^− 05^), MDR(781,687;5.3 × 10^− 05^), RMP(781,687;4.8 × 10^− 04^), STM(781,687;4.1 × 10^− 08^)	4
*rpsL*	STM	4	AG(781,687;3.8 × 10^− 4^), INH(781,687;4.3 × 10^− 15^), MDR(781,687;3.9 × 10^− 12^), PZA(781,687;6.1 × 10^− 06^), RMP(781,687;8.3 × 10^− 10^), STM(781,687;9.6 × 10^− 14^, 781,822;2.3 × 10^− 04^)	6
*rpsL*	STM	2 + 4	AG(781,687;3.8 × 10^− 5^), EMB(781,687;3.5 × 10^− 05^), FQ(781,687;8.3 × 10^− 05^), INH(781,687;2.3 × 10^− 26^, 781,822;6.4 × 10^− 05^), MDR(781,687;2.3 × 10^− 25^, 781,822;4.1 × 10^− 06^), PZA(781,687;1.5 × 10^− 08^), RMP(781,687;4.8 × 10^− 22^, 781,822;8.6 × 10^− 06^), STM(781,687;3.4 × 10^− 30^, 781,822;2.6 × 10^− 07^), XDR(781,687;4.3 × 10^− 09^)	13
*Rv1482c-fabG1*	INH, ETH	2	INH(1,673,425;9 × 10^− 06^), MDR(1,673,425;5.7 × 10^− 05^)	2
*Rv1482c-fabG1*	INH, ETH	4	INH(1,673,425;2.2 × 10^− 20^), MDR(1,673,425;2 × 10^− 07^), XDR(1,673,425;3.9 × 10^− 05^)	3
*Rv1482c-fabG1*	INH, ETH	2 + 4	EMB(1,673,432;5.4 × 10^− 05^), ETH(1,673,425;7.6 × 10^− 04^), FQ(1,673,432;9 × 10^− 04^), INH(1,673,425;6.4 × 10^− 27^, 1,673,432;8.3 × 10^− 07^), MDR(1,673,425;4.4 × 10^− 14^, 1,673,432;6.4 × 10^− 05^), RMP(1,673,432;7.9 × 10^− 06^, 1,673,425;3 × 10^− 05^), STEP(1,673,432;8.5 × 10^− 05^, 1,673,425;3.6 × 10^− 04^), XDR(1,673,425;8.7 × 10^− 06^, 1,673,432;1.2 × 10^− 05^)	14
*gyrA*	FQ	2	EMB(7582;1.3 × 10^− 05^), ETH(7582;9.5 × 10^− 04^), FQ(7582;2.3 × 10^− 09^, 7570;7.6 × 10–06, 7581;4.1 × 10^− 04^), INH(7582;9.9 × 10^− 06^), MDR(7582;7.9 × 10^− 05^), OFL(7582;1.4 × 10^− 06^, 7570;8.5 × 10^− 04^, 7581;8.5 × 10^− 04^), RMP(7582;2.6 × 10^− 06^, 7570;1.2 × 10^− 04^, 7581;9.3 × 10^− 04^), STM(7582;6.5 × 10^− 04^), XDRvMDR(7570;9.6 × 10^− 04^), XDR(7570;5.7 × 10^− 07^, 7582;7.5 × 10^− 07^, 7581;7.5 × 10^− 04^)	21
*gyrA*	FQ	4	EMB(7570;1.2 × 10^− 05^), FQ(7570;1.9 × 10^− 08^, 7582;3 × 10^− 06^, 7581;2.1 × 10^− 05^), INH(7570;3.2 × 10^− 10^, 7581;9.2 × 10^− 05^, 7582;1.1 × 10^− 04^), KAN(7570;1.5 × 10^− 04^), MDR(7570;1 × 10^− 08^, 7582;4.2 × 10^− 06^, 7581;5 × 10^− 05^), OFL(7570;2.5 × 10^− 04^, 7582;5.6 × 10^− 04^), PZA(7570;1 × 10^− 05^, 7581;1.3 × 10^− 04^), RMP(7570;3.4 × 10^− 11^, 7582;5 × 10^− 08^, 7581;5.1 × 10^− 06^), XDR(7570;3.3 × 10^− 10^, 7582;2.7 × 10^− 05^, 7572;3.6 × 10^− 04^)	24
*gyrA*	FQ	2 + 4	AMK(7570;6.5 × 10^− 04^), CAP(7570;9.9 × 10^− 04^), EMB(7582;4.2 × 10^− 13^, 7570;1.1 × 10^− 08^, 7572;3.7 × 10^− 04^, 7581;4.8 × 10^− 04^), ETH(7582;1.3 × 10^− 04^), FQ(7582;3.6 × 10^− 18^, 7570;4.1 × 10^− 14^, 7581;2.4 × 10^− 09^, 7572;6.1 × 10^− 04^), INH(7582;4.5 × 10^− 15^, 7570;4.2 × 10^− 14^, 7581;1.2 × 10^− 08^, 7572;8.3 × 10^− 06^), KAN(7570;5.7 × 10^− 06^, 7572;6.7 × 10^− 05^), MDR(7582;2.2 × 10^− 15^, 7570;1.8 × 10^− 11^, 7581;6.4 × 10^− 07^), OFL(7582;1.3 × 10–10, 7570;2 × 10–07, 7581;5.8 × 10^− 06^), PZA(7581;1.2 × 10^− 07^, 7570;2.4 × 10^− 07^, 7572;9.7 × 10^− 05^, 7582;4.6 × 10^− 04^), RMP(7582;2.3 × 10^− 20^, 7570;2.5 × 10^− 17^, 7581;1.8 × 10–10, 7572;1 × 10^− 06^), STM(7582;1.9 × 10^− 10^, 7570;1.5 × 10^− 06^, 7581;1.8 × 10^− 04^), XDRvMDR(7570;4.2 × 10^− 05^, 7582;5.7 × 10^− 04^), XDR(7570;3.4 × 10^− 19^, 7582;3 × 10^− 16^, 7572;9.7 × 10^− 08^, 7581;2.9 × 10^− 07^)	44
*rrs*	STM, AG	2	AMK(1,473,246;1.8 × 10^− 04^), CAP(1,473,246;5 × 10^− 08^), INH(1,473,246;5 × 10^− 06^), KAN(1,473,246;1.3 × 10^− 11^), RMP(1,473,246;8.9 × 10^− 06^), STM(1,473,246;4.1 × 10^− 04^), XDRvMDR(1,473,246;3.6 × 10^− 05^), XDR(1,473,246;7.4 × 10^− 11^)	8
*rrs*	STM, AG	4	AG(1,473,246;2.6 × 10^−7^), AMK(1,473,246;4.6 × 10^− 06^), CAP(1,473,246;2 × 10^− 06^), CIP(1,473,246;9.4 × 10^− 04^), EMB(1,473,246;7.1 × 10^− 07^), FQ(1,473,246;2.5 × 10^− 04^), INH(1,473,246;3.2 × 10^− 10^), KAN(1,473,246;3.3 × 10^− 10^), MDR(1,473,246;4.6 × 10^− 06^), PZA(1,473,246;9.4 × 10^− 10^), RMP(1,473,246;1.9 × 10^− 16^), STM(1,473,246;3.1 × 10^− 05^, 1,472,359;2.3 × 10^− 04^), XDRvMDR(1,473,246;1.9 × 10^− 05^), XDR(1,473,246;1.6 × 10^− 13^)	15
*rrs*	STM, AG	2 + 4	AG(1,473,246;7.5 × 10^−5^), AMK(1,473,246;3.9 × 10^− 11^), CAP(1,473,246;7.2 × 10^− 14^), CIP(1,473,246;6.5 × 10^− 04^), EMB(1,473,246;2.5 × 10^− 11^), FQ(1,473,246;3.5 × 10^− 07^), INH(1,473,246;3.6 × 10^− 20^, 1,472,359;1.2 × 10^− 05^), KAN(1,473,246;7.9 × 10^− 22^), MDR(1,473,246;1.8 × 10^− 11^, 1,472,359;4.4 × 10^− 04^), PZA(1,473,246;2.6 × 10^− 10^), RMP(1,473,246;7.3 × 10^− 26^), STM(1,473,246;1.3 × 10^− 11^, 1,472,359;1.5 × 10^− 08^), XDRvMDR(1,473,246;2.1 × 10^− 09^), XDR(1,473,246;7.9 × 10^− 29^, 1,472,359;1.5 × 10^− 04^)	18
*thyX-hsdS.1*	PAS	2	XDR(3,067,961;7.5 × 10^−04^)	1
*thyX-hsdS.1*	PAS	4	INH(3,067,961;4.9 × 10^−04^), STM(3,067,961;3.2 × 10^− 04^)	2
*thyX-hsdS.1*	PAS	2 + 4	EMB(3,067,961;1 × 10^− 05^), INH(3,067,961;1.4 × 10^− 07^), MDR(3,067,961;6.4 × 10^− 05^), RMP(3,067,961;9.4 × 10^− 05^), STM(3,067,961;2.3 × 10^− 07^), XDR(3,067,961;1.2 × 10^− 05^)	6
*rpoC*	RMP	4	EMB(764,817;2 × 10^− 04^), MDR(764,817;6 × 10^− 07^, 764,840;2.8 × 10^− 04^), PZA(764,817;8.3 × 10^− 08^), RMP(764,817;3.6 × 10^− 08^, 764,840;4.9 × 10^− 04^, 767,123;4.9 × 10^− 04^), STM(764,817;3.4 × 10^− 04^)	8
*rpoC*	RMP	2 + 4	EMB(764,817;1.9 × 10^− 04^), INH(764,817;1.1 × 10^− 05^, 764,840;1.1 × 10^− 04^), MDR(764,817;4.9 × 10^− 10^, 764,840;9.7 × 10^− 06^), PZA(764,817;2 × 10^− 07^), RMP(764,817;1.4 × 10^− 09^, 764,840;2.2 × 10^− 05^, 764,363;4.7 × 10^− 04^, 767,123;4.7 × 10^− 04^), STM(764,817;3.8 × 10^− 06^)	11
*embC-embA*	EMB	2	EMB(4,243,217;1.7 × 10^−04^)	1
*embC-embA*	EMB	4	EMB(4,243,221;3.7 × 10^−04^, 4,243,190;3.8 × 10–04), INH(4,243,221;8.5 × 10^− 05^), MDR(4,243,217;4.6 × 10^− 06^, 4,243,221;4.6 × 10^− 06^, 4,243,190;3.6 × 10^− 05^), RMP(4,243,221;1.1 × 10^− 05^, 4,243,190;4.9 × 10^− 04^)	8
*embC-embA*	EMB	2 + 4	EMB(4,243,217;1.4 × 10^−07^, 4,243,190;3 × 10^− 07^, 4,243,221;1.3 × 10^− 06^), INH(4,243,217;4 × 10^− 08^, 4,243,221;2.3 × 10^− 06^, 4,243,190;3 × 10^− 05^), MDR(4,243,217;1.2 × 10^− 09^, 4,243,221;6.9 × 10^− 08^, 4,243,190;1.9 × 10^− 06^), RMP(4,243,221;2.2 × 10^− 07^, 4,243,217;6.1 × 10^− 07^, 4,243,190;4.8 × 10^− 06^), STM(4,243,217;8.6 × 10^− 04^)	13
***hadA***	Novel	4	INH(732,110;4 × 10^−04^), MDR(732,110;2.8 × 10^− 04^)	2
***hadA***	Novel	2 + 4	INH(732,110;1.1 × 10^−04^), MDR(732,110;2.6 × 10^− 04^), STM(732,110;4 × 10^− 04^)	3
*pncA*	PZA	4	EMB(2,288,868;3.8 × 10^− 04^), MDR(2,288,764;2.8 × 10^− 04^), RMP(2,288,764;4.9 × 10^− 04^)	3
*pncA*	PZA	2 + 4	EMB(2,288,820;1.9 × 10^− 04^, 2,289,103;1.9 × 10^− 04^), MDR(2,289,207;2.6 × 10^− 04^), PZA(2,289,207;9.7 × 10^− 05^), RMP(2,288,778;4.7 × 10^− 04^, 2,288,820;4.7 × 10^− 04^)	6
*pncA-Rv2044c*	PZA	4	RMP(2,289,252;4.9 × 10^− 04^), XDR(2,289,252;3.6 × 10^− 04^)	2
*pncA-Rv2044c*	PZA	2 + 4	INH(2,289,252;1.1 × 10^− 04^), MDR(2,289,252;5 × 10^− 05^), PZA(2,289,252;2 × 10^− 07^), RMP(2,289,252;4.8 × 10^− 06^), XDR(2,289,252;6.2 × 10^− 05^)	5
***Rv3115-moeB2***	Novel	2 + 4	MDR(3,482,717;6.7 × 10^− 04^), STM(3,482,717;6.7 × 10^− 04^)	2
*eis-Rv2417c*	AG	2 + 4	EMB(2,715,342;1.6 × 10^− 05^), FQ(2,715,342;1.7 × 10^− 04^), INH(2,715,342;1.1 × 10^− 04^), KAN(2,715,342;5.4 × 10^− 04^), RMP(2,715,342;2.2 × 10^− 05^), STM(2,715,342;1.4 × 10^− 05^)	7
*folC*	PAS	4	EMB(2,747,471;3.8 × 10^−04^)	1
*folC*	PAS	2 + 4	EMB(2,747,471;3.7 × 10^−04^), INH(2,747,471;1.1 × 10^− 04^), STM(2,747,471;4 × 10^− 04^)	3
*whiB6-Rv3863*	Putative STM or ETH	2 + 4	EMB(4,338,594;9 × 10^− 04^)	1
*fabG1*	INH [53]	4	INH(1,674,048;6.3 × 10^− 06^)	1
*fabG1*	INH [53]	2 + 4	INH(1,674,048;5.5 × 10^− 06^)	1
*oxyR’-ahpC*	INH	2 + 4	INH(2,726,141;6.8 × 10^− 04^), PZA(2,726,141;9 × 10^− 04^)	2
*gyrB*	FQ	4	RMP(6620;4.9 × 10^− 04^)	1
*gyrB*	FQ	2 + 4	RMP(6620;4.7 × 10^−04^)	1

(*p-value*s < 1E-3) Drug resistance phenotype abbreviations are as given in methods. ‘Total’ refers to the total number of significantly associated variants for the locus and lineage in question. AMK = Amikacin-resistance, AG = Aminoglycoside-resistance, CAP = Capreomycin-resistance, CIP = Ciprofloxacin-resistance, EMB = Ethambutol-resistance, ETH = Ethionamide-resistance, FQ = Fluoroquinolone-resistance, INH = Isoniazid-resistance, KAN = Kanamycin-resistance, MDR = Multidrug-resistant, OFL = Ofloxacin-resistance, PAN = pan-susceptible (no known drug-resistance), PZA = Pyrazinamide-resistance, RMP = Rifampicin-resistance, STM = Streptomycin-resistance, XDR = Extensively drug-resistant

**Fig. 2:**
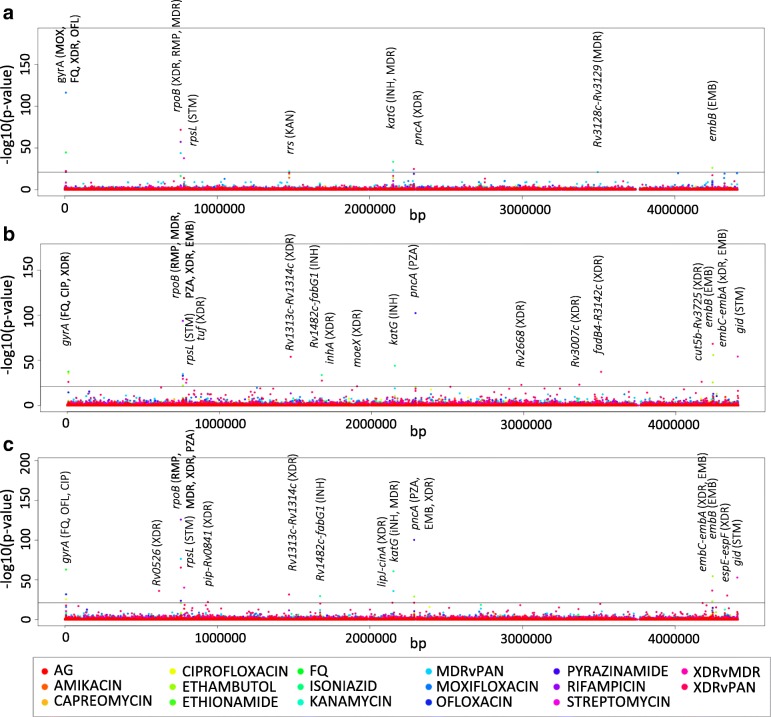
Locus-based GWAS results **a** Manhattan plot for locus-based GWAS for lineage 2. **b** Manhattan plot for locus-based GWAS for lineage 4 **c** Manhattan plot for locus-based GWAS for lineages 2 and 4 combined. *P-value* threshold <1E-21. Phenotypes with which loci were found to be significantly associated are given in brackets next to locus name. AMK = Amikacin-resistance, AG = Aminoglycoside-resistance, CAP = Capreomycin-resistance, CIP = Ciprofloxacin-resistance, EMB = Ethambutol-resistance, ETH = Ethionamide-resistance, FQ = Fluoroquinolone-resistance, INH = Isoniazid-resistance, KAN = Kanamycin-resistance, MDR = Multidrug-resistant, OFL = Ofloxacin-resistance, PAN = pan-susceptible (no known drug-resistance), PZA = Pyrazinamide-resistance, RMP = Rifampicin-resistance, STM = Streptomycin-resistance, XDR = Extensively drug-resistant

**Fig. 3:**
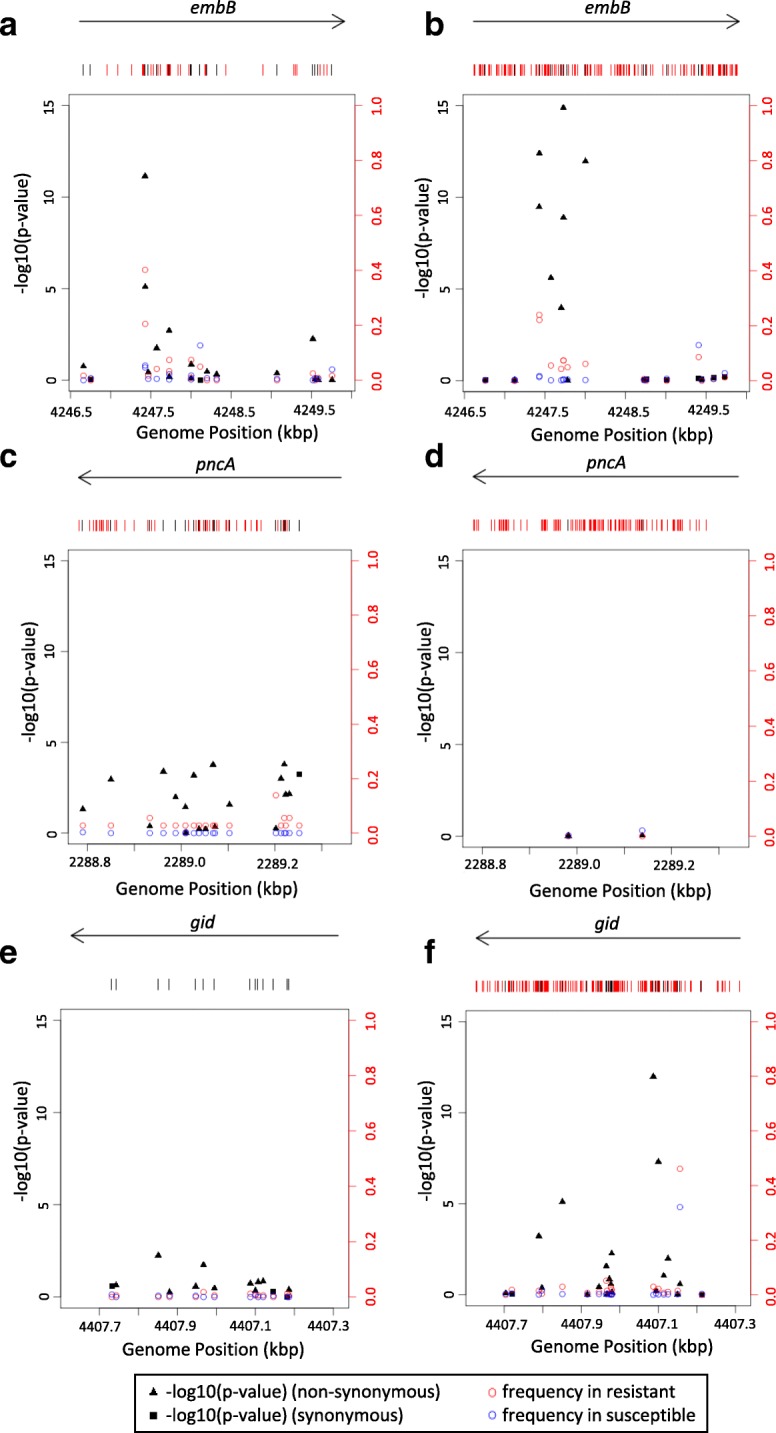
Locus maps showing variant-based GWAS results (on the left y-axis) and variant frequency (on the right y-axis): **a** lineage 2 ethambutol analysis for *embB*; **b** lineage 4 ethambutol analysis for *embB;* **c** lineage2 XDR analysis for *pncA;* **d** lineage 4 XDR analysis for *pncA;* **e** lineage 2 streptomycin analysis for *gid;* **f** lineage 4 streptomycin analysis for *gid*

Locus-based GWAS identified 23 different loci (see Table 2**,** Fig. 2**,** Additional file 7). Fourteen such loci were identified by locus-based GWAS exclusively; of these 14 loci, *gid* is known to be involved in streptomycin resistance and *inhA* is known to be involved in isoniazid and ethionamide resistance [30, 31] (see Additional file 8). Variant-based GWAS identified eleven variants in nine different loci. No known associations were identified by variant-based GWAS exclusively; however, three novel associations were identified (*RV0197, recF, argJ*) (see Table 3**,** Additional file 8). Three loci were identified by locus-based GWAS and PhyC but not variant-based GWAS: *pncA* (pyrazinamide), *embC-embA* and *embB* (ethambutol) (see Fig. 3a and b**,** Additional file 8).

**Table 2:** Significant associations between loci and drug resistance phenotypes identified by locus-based GWAS

Locus	Known Phenotype Association [13]	Lineage	Observed Phenotype Association	P-value	PhyC
*rpoB*	RMP	2	XDR, RMP, MDR	1.9 × 10^− 72^, 5.2 × 10^− 58^, 1.4 × 10^− 44^	11
*rpoB*	RMP	4	RMP, MDR, PZA, XDR, EMB	2 × 10^− 94^, 1.1 × 10^− 35^, 1.1 × 10^− 33^, 2.5 × 10^− 30^, 6.4 × 10^− 23^	23
*rpoB*	RMP	2 + 4	RMP, MDR, XDR, PZA	1.6 × 10^− 126^, 4.1 × 10^− 77^, 4.1 × 10^− 66^, 2.1 × 10^− 24^	40
*gyrA*	FQ	2	MOX, FQ, XDR, OFL	5.2 × 10^− 117^, 2.5 × 10^− 45^, 4.3 × 10^− 23^, 5.1 × 10^− 22^	21
*gyrA*	FQ	4	FQ, CIP, XDR	4.1 × 10^− 38^, 5.4 × 10^− 36^, 6.5 × 10^− 27^	24
*gyrA*	FQ	2 + 4	FQ, OFL, CIP	1.2 × 10^− 63^, 1.7 × 10^− 32^, 2.2 × 10^− 26^	44
*pncA*	PZA	2	XDR	1.50 × 10^− 25^	0
*pncA*	PZA	4	PZA	4.50 × 10^− 103^	3
*pncA*	PZA	2 + 4	PZA, EMB, XDR	5.3 × 10^− 101^, 1.2 × 10^− 29^, 6.7 × 10^− 22^	6
*embC-embA*	EMB	4	XDR, EMB	6.1 × 10^− 69^, 2.8 × 10^− 26^	8
*embC-embA*	EMB	2 + 4	XDR, EMB	3.3 × 10^− 37^, 1.4 × 10^− 23^	13
*katG*	INH	2	INH, MDR	3.6 × 10^−34^, 7.3 × 10^−24^	5
*katG*	INH	4	INH	1.20 × 10^−44^	9
*katG*	INH	2 + 4	INH, MDR	1.5 × 10^−61^, 1.5 × 10^−36^	9
*embB*	EMB	2	EMB	7.20 × 10^−27^	14
*embB*	EMB	4	EMB	1.80 × 10^−56^	31
*embB*	EMB	2 + 4	EMB	3.30 × 10^−55^	41
*gid*	STM	4	STM	7.40 × 10^− 55^	0
*gid*	STM	2 + 4	STM	1.30 × 10^−53^	0
***Rv1313c-Rv1314c***		4	XDR	1.40 × 10^−54^	0
***Rv1313c-Rv1314c***		2 + 4	XDR	3.30 × 10^−32^	0
*rpsL*	STM	2	STM	1.90 × 10^−38^	4
*rpsL*	STM	4	STM	5.60 × 10^−26^	6
*rpsL*	STM	2 + 4	STM	6.00 × 10^−41^	13
***fadB4-Rv3142c***		4	XDR	4.60 × 10^−38^	0
***Rv0526***		2 + 4	XDR	8.70 × 10^−37^	0
*Rv1482c-fabG1*	INH, ETH	4	INH	1.70 × 10^−34^	3
*Rv1482c-fabG1*	INH, ETH	2 + 4	INH	3.30 × 10^−30^	14
***espE-espF***		2 + 4	XDR	5.70 × 10^−31^	0
***tuf***		4	XDR	1.50 × 10^−29^	0
*inhA*	INH, ETH	4	XDR	2.40 × 10^−28^	0
***cut5b-Rv3725***		4	XDR	5.10 × 10^−27^	0
***Rv3007c***		4	XDR	7.80 × 10^−24^	0
***Rv2668***		4	XDR	1.30 × 10^−23^	0
***pip-Rv0841***		2 + 4	XDR	8.60 × 10^− 23^	0
*rrs*	STM, AG	2	KAN	1.40 × 10^−22^	8
***moeX***		4	XDR	5.50 × 10^− 22^	0
***lipJ-cinA***		2 + 4	XDR	6.20 × 10^−22^	0
***Rv3128c-Rv3129***		2	MDR	7.40 × 10^−22^	0

(P-values <1E-21) Novel associations are given in bold. ‘PhyC’ column refers to the number of associations identified through PhyC analysis for the locus and lineage in question. AMK = Amikacin-resistance, AG = Aminoglycoside-resistance, CAP = Capreomycin-resistance, CIP = Ciprofloxacin-resistance, EMB = Ethambutol-resistance, ETH = Ethionamide-resistance, FQ = Fluoroquinolone-resistance, INH = Isoniazid-resistance, KAN = Kanamycin-resistance, MDR = Multidrug-resistant, OFL = Ofloxacin-resistance, PAN = pan-susceptible (no known drug-resistance), PZA = Pyrazinamide-resistance, RMP = Rifampicin-resistance, STM = Streptomycin-resistance, XDR = Extensively drug-resistant

**Table 3:** Significant associations between genomic variants and drug resistance phenotypes identified by variant-based GWAS

Variant Locus	Variant Position	Type	Known Phenotype Association [13]	Lineage	Observed Phenotype Association (*p-value*)	PhyC
*rrs*	1,473,246	inter	STM, AG	2	CAP(2 × 10^−31^), KAN(1.1 × 10^−37^)	8
*rrs*	1,473,246	inter	STM, AG	4	KAN(6.7 × 10^−31^)	15
*rrs*	1,473,246	inter	STM, AG	2 + 4	AMK(2.4 × 10^−39^), CAP(3.9 × 10^−48^), KAN(6.5 × 10^−69^), XDRvMDR(5.3 × 10^−27^)	18
*katG*	2,155,168	NS	INH	2	XDR(2.1 × 10^− 42^)	5
*katG*	2,155,168	NS	INH	4	INH(6.1 × 10^−65^), MDR(6 × 10^−45^), XDR(1.5 × 10^−29^)	9
*katG*	2,155,168	NS	INH	2 + 4	INH(4.4 × 10–^56^), MDR(7.4 × 10^−25^)	9
***Rv0197***	232,574	NS	Novel	4	XDR(9.5 × 10^−62^)	0
***Rv0197***	232,574	NS	Novel	2 + 4	XDR(232,574;3.8 × 10^−51^)	0
*rpoB*	761,155	NS	RMP	2	XDR(3.5 × 10^− 25^)	4
*rpoB*	761,155	NS	RMP	4	MDR(1.2 × 10^−27^), PZA(1.9 × 10^−28^), RMP(2.6 × 10^− 42^, 7.1 × 10^−31^, 761,139;3.4 × 10^−23^), XDR(3.8 × 10^− 57^)	7
*rpoB*	761,139	NS	RMP	4	RMP(3.4 × 10^−23^)	3
*rpoB*	761,155	NS	RMP	2 + 4	MDR(5 × 10^−23^), PZA(6 × 10^−26^), RMP(2 × 10^−38^), XDR(1.3 × 10^− 27^)	7
*rpoB*	761,139	NS	RMP	2 + 4	PZA(4 × 10^− 23^), RMP(2.2 × 10^−29^),	7
***recF***	4047	S	Novel	4	XDR(1.2 × 10^− 52^)	0
***recF***	4047	S	Novel	2 + 4	XDR(8.6 × 10^−41^)	0
*Rv1482c-fabG1*	1,673,425	inter	INH, ETH	4	INH(1.1 × 10^−36^)	3
*Rv1482c-fabG1*	1,673,425	inter	INH, ETH	2 + 4	INH(1.1 × 10^− 35^)	14
*rpsL*	781,687	NS	STM	2	STM(3 × 10^−27^)	4
*rpsL*	781,687	NS	STM	2 + 4	STM(6.3 × 10^−28^)	6
***argJ***	1,867,614	S	Novel	2 + 4	XDR(6.9 × 10^−26^)	0
*gyrA*	7570	NS	FQ	4	XDR(8.6 × 10^−23^)	24
*gyrA*	7582	NS	FQ	2 + 4	CIP(1.3 × 10^−24^), FQ(4.6 × 10^−22^)	44

(*p-value*s < 1E-22) NS = non-synonymous, S = synonymous, inter = intergenic region. Novel associations are given in bold. ‘PhyC’ column refers to the number of associations identified through PhyC analysis for the locus and lineage in question; AMK = Amikacin-resistance, AG = Aminoglycoside-resistance, CAP = Capreomycin-resistance, CIP = Ciprofloxacin-resistance, EMB = Ethambutol-resistance, ETH = Ethionamide-resistance, FQ = Fluoroquinolone-resistance, INH = Isoniazid-resistance, KAN = Kanamycin-resistance, MDR = Multidrug-resistant, OFL = Ofloxacin-resistance, PAN = pan-susceptible (no known drug-resistance), PZA = Pyrazinamide-resistance, RMP = Rifampicin-resistance, STM = Streptomycin-resistance, XDR = Extensively drug-resistant

### Effects of lineage-specific analysis on identifying known resistance associated variants

#### Lineage 2 specific

Overall, for locus-based GWAS analyses across the 16 phenotypes, two loci were identified exclusively to lineage 2 specific analyses; *rrs (KAN; p*-value = 1.40 × 10^− 22^*)* and *Rv3128c-Rv3129* (MDR; p-value = 7.4 × 10^− 22^) (see Fig. 2a). For locus-based GWAS, *pncA* was found in association with XDR exclusively, however for lineage 4 *pncA* was found in association with PZA exclusively; greater variation was found in the *pncA* locus for lineage 2 (see Fig. 3c and d). For the variant-based GWAS analyses there were no lineage 2 exclusive associations. Furthermore, no lineage 2 exclusive associations were identified by PhyC analyses.

#### Lineage 4 specific

Overall, for the locus-based GWAS analyses, seven loci were identified exclusively by lineage 4 specific analyses (*inhA, fadB4-Rv3142c*, *tuf*, *cut5b-Rv3725*, *Rv3007c*, *Rv2668*, *moeX*) (see Fig. 2b). All of which were found in significant association with the XDR phenotype. For locus-based GWAS, *gid* was identified in association with streptomycin by lineage 4 specific analyses and –combined analyses but not lineage 2 specific analyses; there is greater variation within the *gid* locus for lineage 4 (see Fig. 3e and f**)**. The variant-based GWAS analyses identified no lineage 4 exclusive analyses. Moreover, no lineage 4 exclusive associations were identified by PhyC analyses.

#### Lineages 2 and 4 combined

Four loci were solely identified through combined lineage PhyC analyses; *Rv3115-moeB2* (MDR, STM; min. *p*-value = 6.7 × 10^− 4^)*, eis-Rv2417c* (STM; min. p-value = 1.4 × 10^− 05^)*, whib6-Rv3863* (EMB; p-value = 9 × 10^− 4^) and *oxyR’-ahpC* (INH, PZA; *p*-values = 6.8 × 10^− 4^, 9 × 10^− 4^, respectively) (see Table 1). For each loci identified by PhyC, there were consistently the same number or more associations identified by the -combined versus the lineage-specific analyses (see Table 1).

For locus-based GWAS, four loci were identified in association with XDR by the combined lineages 2 and 4 analyses exclusively; *Rv0526* (*p*-value = 8.70 × 10^− 37^; thioredoxin protein) and three intergenic regions; *espE-espF* (*p*-value = 5.70 × 10^− 31^)*, pip-Rv0841* (p-value = 8.60 × 10^− 23^) and *lipJ-cinA* (*p*-value = 6.20 × 10^− 22^) (see Table 2**,** Fig. 2c)*.*

For variant-based GWAS, one locus was identified by the combined lineages 2 and 4 analyses exclusively; *argJ*, in association with XDR (*p*-value = 6.9 × 10^− 26^) (see Table 3).

### Novel resistance-associated variants identified

Across all analyses, we report 17 potentially novel associations between antimicrobial resistance and genomic variants in *Mtb;* 7 such associations were identified exclusively by lineage-specific analyses (see Tables 1, 2, 3). Twelve were identified by locus-based GWAS, three were identified by variant-based GWAS and two were identified by PhyC. All novel associations identified by GWAS were found in association with the XDR phenotype. There was no overlap in novel associations identified between methods.

Two potentially novel associations were identified by PhyC; *hadA* (lineage 4, 2 + 4; INH, MDR and STM; 1.1 × 10^− 4^ < *p*-values< 4 × 10^− 4^) and *Rv3115-moeB2* (lineages 2 + 4; MDR; STM, min. p-value = 6.7 × 10^− 4^) (see Table 1**,** Fig. 1). The *Rv3115-moeB2* variant displays a different pattern of variation within lineage 2 than within lineage 4 (see Fig. 1).

Twelve potentially novel associations were identified by locus-based GWAS (see Table 1). Six loci were identified exclusively in lineage 4 all in association with XDR; *fadB4-Rv3142c* (p-value = 4.6 × 10^− 38^), *tuf* (p-value = 1.5 × 10^− 29^), *Rv3007c* (p-value = 7.8 × 10^− 24^), *cut5b-Rv3725* (p-value = 5.1 × 10^− 27^), *Rv2668* (p-value = 1.3 × 10^− 23^) and *moeX* (p-value = 5.5 × 10^− 22^). *Rv1313c-Rv1314c* was identified by both lineage 4 and lineage-combined analyses in association with XDR (min. p-value = 1.4 × 10^− 54^). Four loci were identified exclusively by lineage-combined analyses in association with XDR; *Rv0526* (p-value = 8.7 × 10^− 37^), *espE-espF* (p-value = 5.7 × 10^− 31^), *pip-Rv0841* (p-value = 8.6 × 10^− 23^) and *lipJ-cinA* (p-value = 6.2 × 10^− 22^). *Rv3128c-Rv3129* was identified exclusively by the lineage 2 analysis in association with MDR (p-value = 7.4 × 10^− 22^) (see Table 2**,** Fig. 1).

Three potentially novel associations were identified by variant-based GWAS, all in association with XDR; in the *Rv0197* locus (lineage 4, 2 + 4; min. p-value = 9.5 × 10^− 62^), in the *recF* locus (lineage 4, 2 + 4; min. p-value = 1.2 × 10^− 52^, respectively) and the *argJ* locus (lineages 2 + 4; p-value = 6.9 × 10^− 26^) (see Table 3**,** Fig. 1).

## Discussion

Our results highlight that lineage specific analyses are able to provide new insights into genetic associations with drug resistance phenotypes, despite a smaller sample size than a pan-lineage approach. Lineage specific associations were found within lineage 2, such as the novel association between *Rv3128c-Rv3129* and MDR. We also identified lineage-specific novel associations within lineage 4, such as the association between *fadB4-Rv3142c* and XDR. This indicates biological differences between these lineages with respect to drug resistance and perhaps in evolutionary trajectory. Novel associations specific to combined analyses indicate convergent evolution between lineages 2 and 4 at the same loci, with variant frequency too low for lineage-specific analyses to detect, that would most likely be detected in larger scale combined analyses (as previously described^13^). Lineage-specific GWAS is complementary to lineage-combined approaches, with their application in tandem potentially improving the power to detect *Mtb* genomic variants evolving under differing evolutionary dynamics.

Overall, despite conservative significance thresholds based on permutation, 17 potential novel associations were identified between antimicrobial resistance and *Mtb* loci and thus warrant experimental validation. For GWAS, 15 novel associations were identified, one in relation to the MDR phenotype and 14 in relation to the XDR phenotype; 7 were lineage specific. This might suggest an evolutionary shift amongst XDR strains. It may be feasible to consider XDR as a highly complex phenotype encompassing transmissibility [32]; unless evolution of XDR from pan-susceptible strains frequently happens within one patient, it is likely that XDR strains have gone through numerous cycles of active disease, transmission and treatment within recent history. The fact that many of these associations are lineage specific lends weight to such a hypothesis, suggesting differing evolutionary trajectories between lineages 2 and 4. Genetic drift might contribute to such divergence; there are numerous bottlenecks during the natural infectious cycle for *Mtb*, driven by host immune system, anti-TB drug therapy and transmission [33].

Some of the novel associated variants may be involved directly in drug resistance such as *hadA*, whose gene product, similar to InhA, is involved in fatty acid synthesis type II (FAS-II)) and thus may be involved in isoniazid resistance [34, 35]. One of the novel associated loci, *Rv0197,* identified here by variant-based GWAS in association with XDR, was previously identified through PhyC in association with a transmissibility phenotype [36]. *EspE* was identified by this previous analysis also [36]*,* and it remains possible that the *espE-espF* intergenic region, identified here by locus-based GWAS in association with XDR, may be related by regulation to *espE*. Additionally, both *espE-espF* and *whiB6-Rv3863* have been linked to Esx-1 which has been implicated in virulence regulation. The *WhiB6-Rv3863* intergenic region*,* which was also identified through previous PhyC analyses including our dataset [13]*,* may additionally be linked to the DosR regulon. This regulon is composed of 48 co-regulated genes and is considered essential for persistence of latent *Mtb* [37–40]. Interestingly, the *whiB6-Rv3863* variant identified shows a markedly different distribution between lineages 2 and 4, showing greater frequency in lineage 2 (see Fig. 1).

Apart from *Rv0197*, a further two variant-based GWAS SNPs were identified (*recF* and *argJ*), however both are synonymous variants. These may be examples of background variants ‘hitchhiking’ alongside causal variants, or may play a biological role. Notably, a number of identified loci are potentially involved in molybdenum cofactor biosynthesis; *Rv3115-moeB2, moeX* [41]*,* and *Rv0197 (mycobrowser: Gene Ontology: molybdenum ion binding)* (Mycobrowser)*.* Molybdenum cofactor is found in molybdenum enzymes, which are responsible for a number of functions from dormancy regulation to energy source metabolism [41, 42]. Interestingly, these three loci were each identified by a different analyses type; variant-based GWAS, locus-based GWAS and PhyC, respectively. Functional studies may be useful in providing further insight into the role of variants identified here.

Recognizing that drug resistance phenotypes may be subtly different, depending on the genetic background of the strain, could be important and might relate directly to drug resistance, or to fitness more broadly, such as through increased virulence and transmission. With the recognition of XDR transmission [36, 43], our study suggests that further critical information on lineage and transmission clustering (obtained from the genome sequence) would also be important to determine the full impact of specific mutations, that might lead to further phenotypic descriptions related to transmission, virulence and degree of drug resistance.

The results show the differing evolutionary insights offered by locus- and variant-based GWAS, and convergence-based methodologies. Both variant-based and locus-based GWAS led to unique loci being identified. The *rrs* locus was found in lineage 2 only locus-based GWAS analyses, but for both variant-based GWAS and PhyC analyses, *rrs* was identified in both lineage-specific and lineage-combined analyses. Neutral variation within the *rrs* gene may be diluting the signal from causal drug resistance variants in the lineage 4 locus-based GWAS analysis.

*inhA* was not identified by variant-based GWAS or PhyC, only lineage 4 specific locus-based GWAS. A sub-type of the Portuguese Lisboa (lineage 4) strain is known to have *inhA* markers involved in isoniazid resistance [44], and a different mechanism to other lineages. Whilst *inhA* was not identified by lineage-combined GWAS, it is notable that *Rv1482c-fabG1* and *katG* were; both these loci also play a role in isoniazid resistance, suggesting different mechanisms of resistance to these drugs between lineage 2 and lineage 4.

In cases where drug resistance is driven by rare variants and genetic heterogeneity exists within a single gene, such as in *pncA*, where multiple alleles can cause pyrazinamide resistance, locus-based analyses may be more powerful. Indeed, *pncA* was identified here by locus-based GWAS but not variant-based GWAS. Convergence-based PhyC analysis seems to have greater sensitivity in combined-lineage analyses. Unlike GWAS, the success of PhyC in detecting antimicrobial resistance associated variants is determined by the magnitude of convergent evolution within the *Mtb* population under question [19]. Indeed, there were important differences between the GWAS and PhyC results outlined here. These differences might provide insight into the relative importance of within patient evolution of antimicrobial resistance versus transmission of antimicrobial resistant strains. In instances where a mutation is highly transmissible and consequently increases in frequency with only one or few mutation events, it might be expected that GWAS would be a more powerful analytical tool, due to the lack of convergent-evolution.

It is notable that lineage 2 had a smaller sample size than the lineage 4 dataset, this may contribute to the greater sensitivity in lineage 4 specific analyses. In order to assess the extent to which the lower significance levels in the lineage 2 GWAS were as a result of smaller sample size in comparison to lineage, it would be interesting to repeat the GWAS analyses with a larger and perhaps more geographically spread lineage 2 dataset. Additionally, statistical power is potentially limited in the current analyses by low resolution phenotypic data, with not all drugs tested on all samples, primarily due to second line drugs only being tested where there is multidrug resistance. For example, for lineage 2 there were only 8 resistant and 120 susceptible isolates for moxifloxacin. Despite this, the most significant gene-based GWAS result for lineage 2 was for *gyrA*, identified in relation to moxifloxacin resistance, showing the sensitivity of the method. Nevertheless, to identify variants with smaller effect sizes, increased phenotypic resolution may prove useful. Further work could explore the use of minimum inhibitory concentration values, where available, being incorporated into resistance phenotypes.

## Conclusions

In summary, GWAS and PhyC are sensitive, robust and complementary methodologies in examining evolution of antimicrobial resistance in *Mtb.* Within GWAS analyses, locus-based and variant-based approaches are both useful and complementary, as are lineage-combined and lineage-specific analyses. These different methodological approaches can be used to detect different evolutionary dynamics and thus their similarities and differences are informative. Evidence presented here suggests the importance of lineage- specific paths of evolution towards drug resistance in *Mtb.* It will be interesting to see how methodologies outlined here might apply to other *Mtb* lineages and other pathogen species in an anti-microbial resistance context, or indeed in relation to other phenotypes of interest such as transmissibility.

## Methods

### Isolates, phenotypic methods, sequencing and variant calling

The raw sequence data used here (*n* = 4408) form part of a subset of a larger dataset (*n* = 6465), which represents multiple populations from different geographic areas (see Additional file 9), and is described elsewhere [13]. In particular, only lineages 2 (*n* = 702) and 4 (*n* = 3706) from the larger dataset are used, with additional phenotypic data for the samples collected in Portugal. Drug resistance phenotypes were available for amikacin, capreomycin, ciprofloxacin, ethambutol, ethionamide, isoniazid, kanamycin, moxifloxacin, ofloxacin, pyrazinamide, rifampicin, streptomycin, resistance to any fluoroquinolone; levofloxacin, moxifloxacin, ciprofloxacin or ofloxacin (FQ), resistance to any of the aminoglycosides; kanamycin, amikacin, or streptomycin (AG), combined isoniazid and rifampicin resistance, but not XDR (MDR), MDR plus resistance to a fluoroquinolone (ciprofloxacin, levofloxacin, moxifloxacin) and to a second line injectable (amikacin, kanamycin, capreomycin) (XDR), and pan-susceptible, susceptibility to rifampicin and isoniazid plus no other known resistance (PAN). Isoniazid, rifampicin, ethambutol, streptomycin and pyrazinamide are first-line drugs. Amikacin, capreomycin, ofloxacin, para-aminosalicylic acid, moxifloxacin and cycloserine are second-line drugs. Samples found to be MDR, underwent testing for second-line drugs. Para-aminosalycylic acid, levofloxacin, rifabutin and cycloserine resistance phenotypes were excluded from analyses due to lack of data. Where present, levofloxacin data was used in defining the aggregate phenotypes of FQ; however, there was not enough levofloxacin phenotypic data to use in individual drug-resistance analyses.

All samples underwent Illumina sequencing generating paired-end reads of at least 50 bp with at least 50-fold average genome coverage. The raw sequence data were aligned to the H37Rv reference genome (Genbank accession number: NC_000962.3) using the BWA mem algorithm [45]. The SAMtools/BCFtools [46] and GATK [47] software was used to call SNPs and small insertions or deletions (indels) using default options. The overlapping set of variants from the two algorithms was retained for further analysis. Alleles were additionally called across the whole genome (including SNP sites) using a coverage-based approach [16, 28]. A missing call was assigned if the total depth of coverage at a site did not reach a minimum of 20 reads or none of the four nucleotides accounted for at least 75% of the total coverage. The final dataset consisted of 157,726 SNPs, 2926 insertions and 5998 deletions across the 4408 isolates. Monomorphic variants within each of the three datasets (‘lineage 4-specific’, ‘lineage 2-specific’ and ‘lineages 2 and 4 combined’) were removed.

### Phylogenetic tree and PhyC

Sublineage was assigned based on SNPs (see Additional file 10). PCA was conducted on the pairwise variant distance matrix for lineages separately and combined. A maximum likelihood phylogenetic tree was constructed for the 157,726 SNP sites present in lineages 2 and 4 isolates using ExaML [48] using the standard model and rooted with *M. canettii* as the outgroup. The ITOL v3 tool was used for visualisation [49]. PhyC [19] analysis was performed using an in-house pipeline as described by Phelan et al. (2016) [16] . A significance cut-off of < 10^− 3^ was applied, and this threshold was established based on permutation analysis.

### Association analyses

Genome wide association study (GWAS) analyses were performed using GEMMA software [50]. The general parameters were; default missingness (< 0.05) and a minor allele frequency cut-off of 0.001. Kinship matrices were used to account for relatedness. Analyses were performed based on SNPs and short indels (range: 1 to 70 bp) (“variant-based”); and mutations aggregated over coding and intergenic loci (“locus-based”). For coding loci, only non-synonymous variants were aggregated. A linear mixed model was used for both types of analysis, and a likelihood ratio test was used to assess statistical significant of the variants and loci. Each analysis considered a different drug susceptibility phenotype, namely: amikacin resistant (AMK) vs. non-amikacin resistant, AG resistant vs. non-AG resistant, capreomycin resistant (CAP) vs. non-capreomycin resistant, ciprofloxacin resistant (CIP) vs. non-ciprofloxacin resistance, ethambutol resistant (EMB) vs. non-ethambutol resistant, ethionamide resistant (ETH) vs. non-ethionamide resistant, isoniazid resistant (INH) vs. non-isoniazid resistant, kanamycin resistant (KAN) vs. non-kanamycin resistant, moxifloxacin resistant (MOX) vs. non-moxifloxacin resistant, ofloxacin resistant vs. non-ofloxacin resistant (OFL), pyrazinamide resistant (PZA) vs. non-pyrazinamide resistant, rifampicin resistant (RMP) vs. non-rifampicin resistant, streptomycin (STM) vs. non-streptomycin resistant, FQ vs. non-FQ, MDR vs. PAN (“MDR”), XDR vs. PAN (“XDR”) and XDR vs. MDR (“XDRvMDR”). Analyses were performed with lineage 4 only (*n* = 3706), lineage 2 only (*n* = 701, after removing 1 outlier identified by PCA) and lineages 2 and 4 combined. Analyses were repeated accounting for different numbers of principal components, from 0 to 5, to assess the effects on significance. A significance threshold of < 10^− 21^ based on permutation.

All statistical analyses, including PCA, were performed in R software (r-project.org) and its qqman package [51] was used to construct Manhattan plots and quantile-quantile (qq)-plots. Pairwise variant distance between isolates was calculated in R [52], using absolute distance between isolates including all variants for lineage 2 and lineage 4.

## Additional files

Additional file 1: Variant Summary Tables, Summary tables of variants called in comparison to the H37rv reference, with monomorphic variants removed for each dataset. **a** Total numbers of variants by lineage; **b** Number of variants per sample; **c** Non-reference variant frequency summary; variants called in comparison to the H37rv reference. (PPTX 39 kb)

Additional file 2: Non-reference variant frequency histogram, A histogram showing log10(frequency + 1) of non-reference alleles compared to the H37rv reference for **a** lineage 2 and **b** lineage4. (PPTX 69 kb)

Additional file 3: Population diversity within investigated strains, **a** Principal component 1 (PC1) by principal component 2 (PC2) for lineage 2, The first 10 principal components account for 71.9% of the variation in lineage 2; **b** Distance plot for lineage 2 showing pairwise number of variant differences between samples; **c** Principal component 1 (PC1) by principal component 2 (PC2) for lineage 4, the first 10 principal components account for 88.9% of the variation in lineage 4. **d** Distance plot for lineage 2 showing pairwise number of variant differences between samples. (PPTX 5650 kb)

Additional file 4: Scree plots for the principal component analyses, Scree plots showing the proportion of variation accounted for by the first ten principal components, calculated for the pairwise distances within **a** lineage 4 and **b** lineage 2. (PPTX 142 kb)

Additional file 5: Drug-resistance phenotype frequency table, Drug-resistance phenotype frequency table by lineage. ‘Totals’ shows the number and percentage of each lineage with a known drug-resistance phenotype. (PPTX 45 kb)

Additional file 6: Cross-resistance phenotype table, Cross-Resistance Table upper diagonal shows proportion of samples phenotyped for both vertical and horizontal phenotype, that test positive for vertical phenotype. Diagonal shows number of samples with each phenotype. Lower diagonal shows number of samples with phenotype for both horizontal and vertical phenotype. (PPTX 45 kb)

Additional file 7: Variant Position Table, Table detailing variants at all positions with at least one non-synonymous variant found to be significantly associated with a phenotype in any of the variant-based analyses. (PPTX 52 kb)

Additional file 8: Locus Comparison Table, Locus comparison table showing which analyses and in which lineage each loci was identified. An ‘x’ indicates a locus which was not identified by the method of analysis in question. Loci without a known association with the phenotype are highlighted in bold. (PPTX 44 kb)

Additional file 9: Study frequency table, Study frequency table, showing numbers and percentage of strains from each study by lineage. (PPTX 40 kb)

Additional file 10: Sublineage frequency table, Numbers and percentage by lineage assigned to each sublineage. (PPTX 36 kb)

## Abbreviations

AG: Aminoglycoside-resistance
AMK: Amikacin-resistance
CAP: Capreomycin-resistance
CIP: Ciprofloxacin-resistance
EMB: Ethambutol-resistance
ETH: Ethionamide-resistance
FQ: Fluoroquinolone-resistance
GWAS: Genome-wide Association Study
INH: Isoniazid-resistance
KAN: Kanamycin-resistance
MDR: Multidrug-resistant
MOX: Moxifloxacin-resistance
OFL: Ofloxacin-resistance
PAN: Pan-susceptible; no known drug-resistance
PAS: Para-Aminosalicylic Acid-resistance
PCA: Principal Component Analysis
PZA: Pyrazinamide-resistance
RMP: Rifampicin-resistance
STM: Streptomycin-resistance
XDR: Extensively drug-resistant
