# Supplementary material for: Genome-wide analysis of Mycobacterium tuberculosis polymorphisms reveals lineage-specific associations with drug resistance
Source: BMC Genomics. 2019 Mar 29;20:252. doi: 10.1186/s12864-019-5615-3 (PMC6440112; doi:10.1186/s12864-019-5615-3)
Supplement: Supplementary file 1 — Variant Summary Tables, Summary tables of variants called in comparison to the H37rv reference, with monomorphic variants removed for each dataset. a Total numbers of variants by lineage; b Number of variants per sample; c Non-reference variant frequency summary; variants called in comparison to the H37rv reference. (PPTX 39 kb) [file 12864_2019_5615_MOESM1_ESM.pptx]

## Slide 1
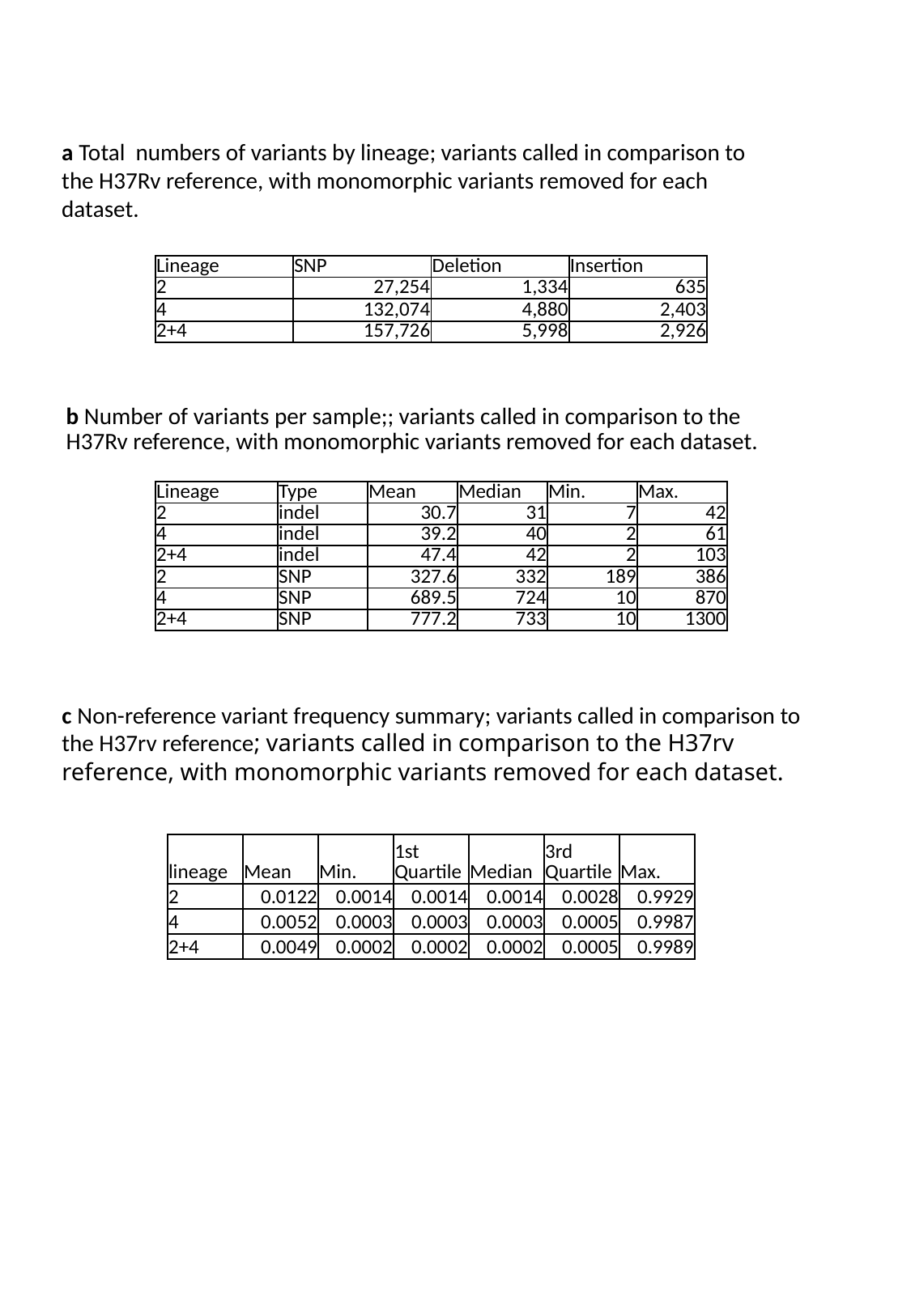

a Total numbers of variants by lineage; variants called in comparison to the H37Rv reference, with monomorphic variants removed for each dataset.
| Lineage | SNP | Deletion | Insertion |
| --- | --- | --- | --- |
| 2 | 27,254 | 1,334 | 635 |
| 4 | 132,074 | 4,880 | 2,403 |
| 2+4 | 157,726 | 5,998 | 2,926 |
b Number of variants per sample;; variants called in comparison to the H37Rv reference, with monomorphic variants removed for each dataset.
| Lineage | Type | Mean | Median | Min. | Max. |
| --- | --- | --- | --- | --- | --- |
| 2 | indel | 30.7 | 31 | 7 | 42 |
| 4 | indel | 39.2 | 40 | 2 | 61 |
| 2+4 | indel | 47.4 | 42 | 2 | 103 |
| 2 | SNP | 327.6 | 332 | 189 | 386 |
| 4 | SNP | 689.5 | 724 | 10 | 870 |
| 2+4 | SNP | 777.2 | 733 | 10 | 1300 |
# c Non-reference variant frequency summary; variants called in comparison to the H37rv reference; variants called in comparison to the H37rv reference, with monomorphic variants removed for each dataset.
| lineage | Mean | Min. | 1st Quartile | Median | 3rd Quartile | Max. |
| --- | --- | --- | --- | --- | --- | --- |
| 2 | 0.0122 | 0.0014 | 0.0014 | 0.0014 | 0.0028 | 0.9929 |
| 4 | 0.0052 | 0.0003 | 0.0003 | 0.0003 | 0.0005 | 0.9987 |
| 2+4 | 0.0049 | 0.0002 | 0.0002 | 0.0002 | 0.0005 | 0.9989 |
