# Supplementary material for: Genome-wide analysis of Mycobacterium tuberculosis polymorphisms reveals lineage-specific associations with drug resistance
Source: BMC Genomics. 2019 Mar 29;20:252. doi: 10.1186/s12864-019-5615-3 (PMC6440112; doi:10.1186/s12864-019-5615-3)
Supplement: Supplementary file 2 — Non-reference variant frequency histogram, A histogram showing log10(frequency + 1) of non-reference alleles compared to the H37rv reference for a lineage 2 and b lineage4. (PPTX 69 kb) [file 12864_2019_5615_MOESM2_ESM.pptx]

## Slide 1
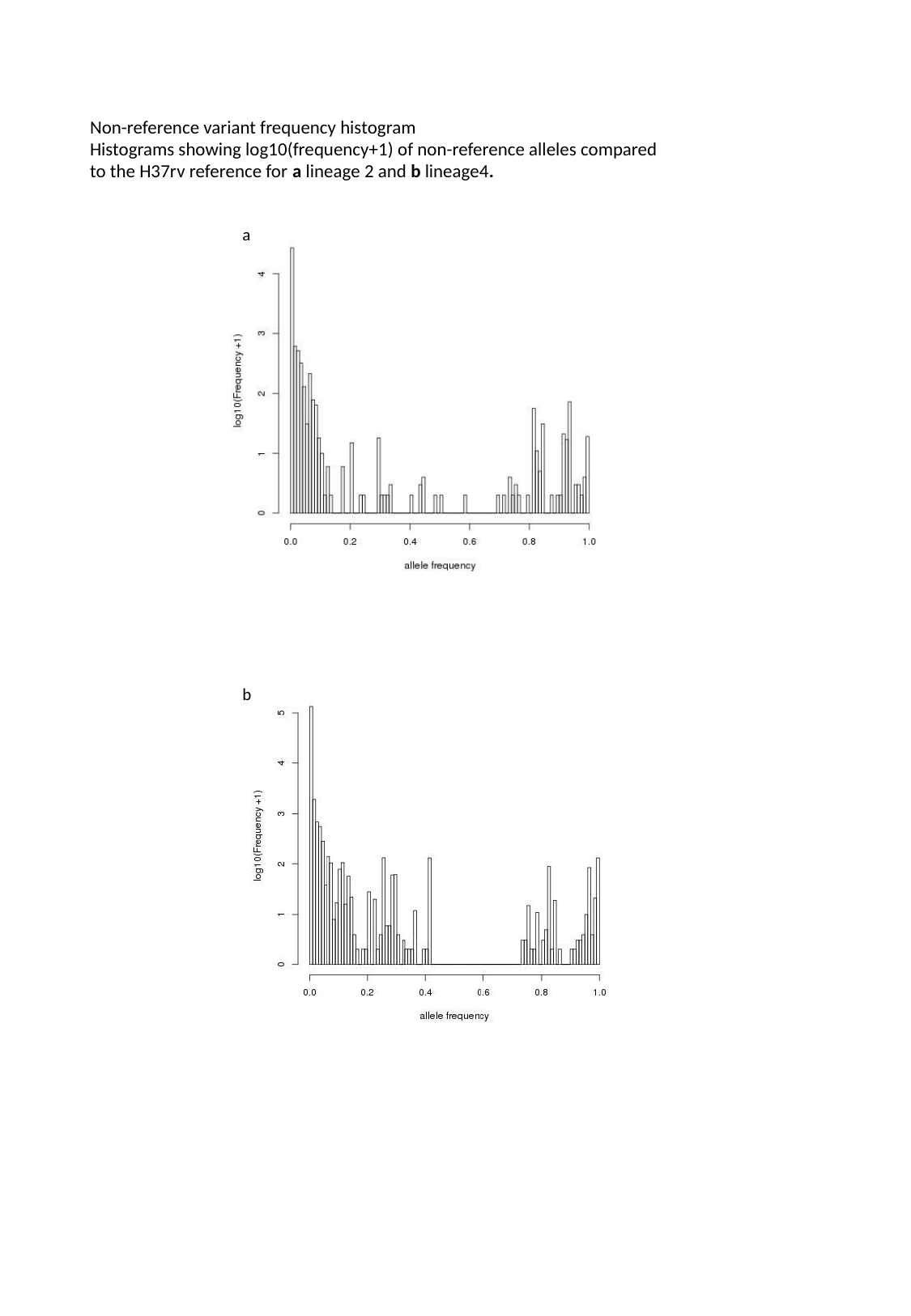

Non-reference variant frequency histogram
Histograms showing log10(frequency+1) of non-reference alleles compared to the H37rv reference for a lineage 2 and b lineage4.
a
b
