# Supplementary material for: Genome-wide analysis of Mycobacterium tuberculosis polymorphisms reveals lineage-specific associations with drug resistance
Source: BMC Genomics. 2019 Mar 29;20:252. doi: 10.1186/s12864-019-5615-3 (PMC6440112; doi:10.1186/s12864-019-5615-3)
Supplement: Supplementary file 3 — Population diversity within investigated strains, a Principal component 1 (PC1) by principal component 2 (PC2) for lineage 2, The first 10 principal components account for 71.9% of the variation in lineage 2; b Distance plot for lineage 2 showing pairwise number of variant differences between samples; c Principal component 1 (PC1) by principal component 2 (PC2) for lineage 4, the first 10 principal components account for 88.9% of the variation in lineage 4. d Distance plot for lineage 2 showing pairwise number of variant differences between samples. (PPTX 5650 kb) [file 12864_2019_5615_MOESM3_ESM.pptx]

## Slide 1
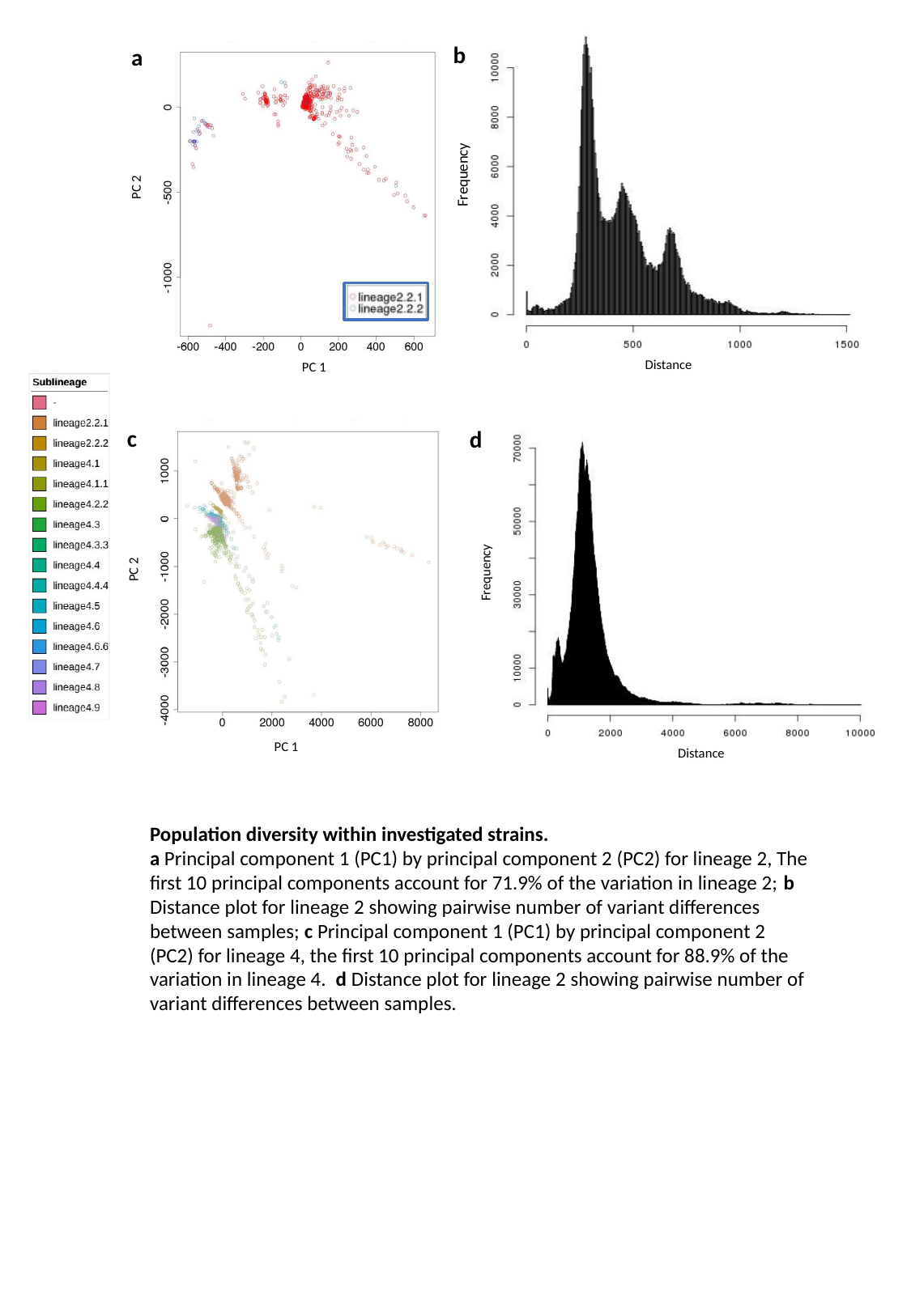

b
a
PC 2
Frequency
Distance
PC 1
c
PC 2
Frequency
PC 1
Distance
d
Population diversity within investigated strains.
a Principal component 1 (PC1) by principal component 2 (PC2) for lineage 2, The first 10 principal components account for 71.9% of the variation in lineage 2; b Distance plot for lineage 2 showing pairwise number of variant differences between samples; c Principal component 1 (PC1) by principal component 2 (PC2) for lineage 4, the first 10 principal components account for 88.9% of the variation in lineage 4. d Distance plot for lineage 2 showing pairwise number of variant differences between samples.
