# Supplementary material for: Genome-wide analysis of Mycobacterium tuberculosis polymorphisms reveals lineage-specific associations with drug resistance
Source: BMC Genomics. 2019 Mar 29;20:252. doi: 10.1186/s12864-019-5615-3 (PMC6440112; doi:10.1186/s12864-019-5615-3)
Supplement: Supplementary file 4 — Scree plots for the principal component analyses, Scree plots showing the proportion of variation accounted for by the first ten principal components, calculated for the pairwise distances within a lineage 4 and b lineage 2. (PPTX 142 kb) [file 12864_2019_5615_MOESM4_ESM.pptx]

## Slide 1
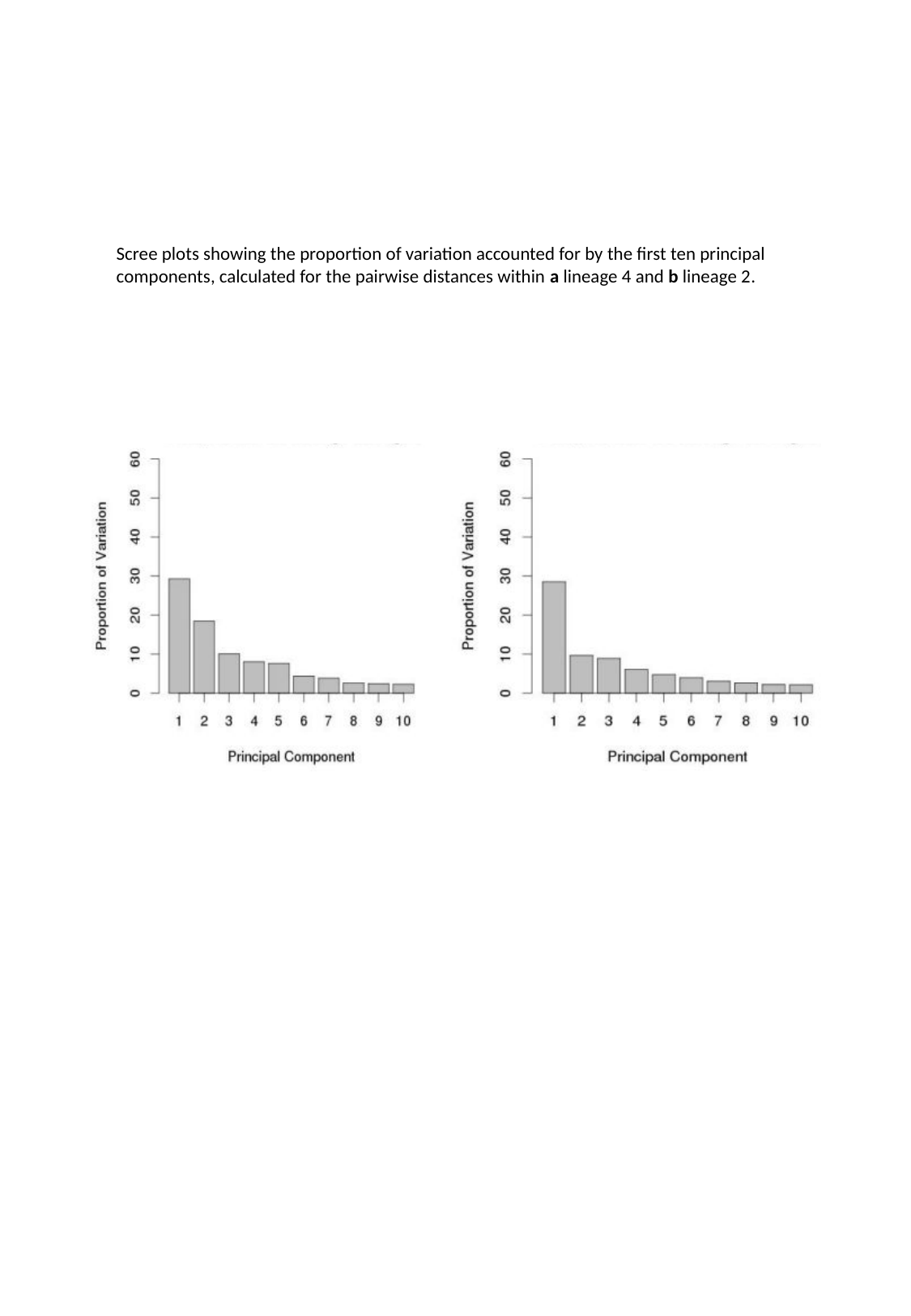

Scree plots showing the proportion of variation accounted for by the first ten principal components, calculated for the pairwise distances within a lineage 4 and b lineage 2.
