# Supplementary material for: Genome-wide analysis of Mycobacterium tuberculosis polymorphisms reveals lineage-specific associations with drug resistance
Source: BMC Genomics. 2019 Mar 29;20:252. doi: 10.1186/s12864-019-5615-3 (PMC6440112; doi:10.1186/s12864-019-5615-3)
Supplement: Supplementary file 5 — Drug-resistance phenotype frequency table, Drug-resistance phenotype frequency table by lineage. ‘Totals’ shows the number and percentage of each lineage with a known drug-resistance phenotype. (PPTX 45 kb) [file 12864_2019_5615_MOESM5_ESM.pptx]

## Slide 1
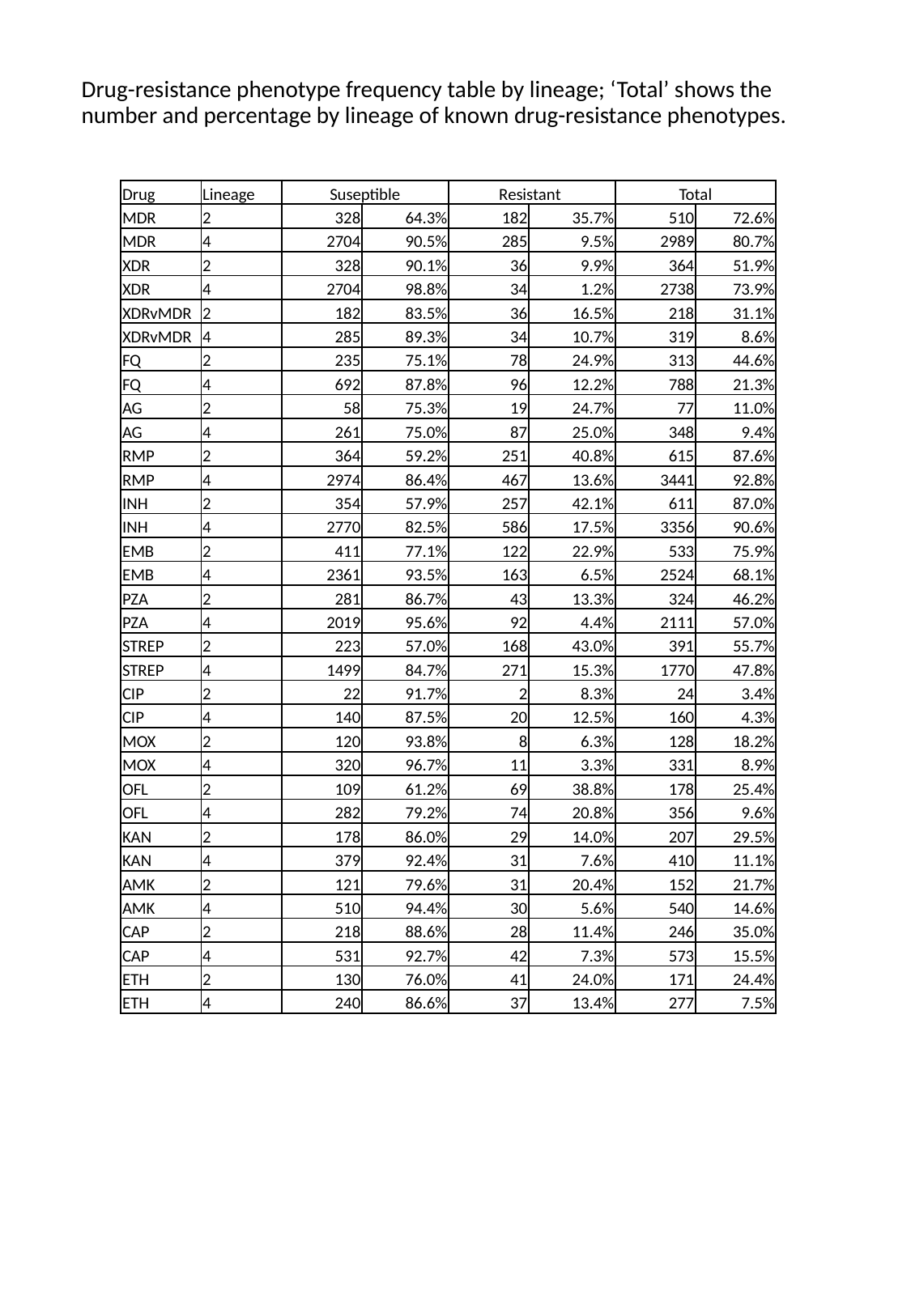

Drug-resistance phenotype frequency table by lineage; ‘Total’ shows the number and percentage by lineage of known drug-resistance phenotypes.
| Drug | Lineage | Suseptible | | Resistant | | Total | |
| --- | --- | --- | --- | --- | --- | --- | --- |
| MDR | 2 | 328 | 64.3% | 182 | 35.7% | 510 | 72.6% |
| MDR | 4 | 2704 | 90.5% | 285 | 9.5% | 2989 | 80.7% |
| XDR | 2 | 328 | 90.1% | 36 | 9.9% | 364 | 51.9% |
| XDR | 4 | 2704 | 98.8% | 34 | 1.2% | 2738 | 73.9% |
| XDRvMDR | 2 | 182 | 83.5% | 36 | 16.5% | 218 | 31.1% |
| XDRvMDR | 4 | 285 | 89.3% | 34 | 10.7% | 319 | 8.6% |
| FQ | 2 | 235 | 75.1% | 78 | 24.9% | 313 | 44.6% |
| FQ | 4 | 692 | 87.8% | 96 | 12.2% | 788 | 21.3% |
| AG | 2 | 58 | 75.3% | 19 | 24.7% | 77 | 11.0% |
| AG | 4 | 261 | 75.0% | 87 | 25.0% | 348 | 9.4% |
| RMP | 2 | 364 | 59.2% | 251 | 40.8% | 615 | 87.6% |
| RMP | 4 | 2974 | 86.4% | 467 | 13.6% | 3441 | 92.8% |
| INH | 2 | 354 | 57.9% | 257 | 42.1% | 611 | 87.0% |
| INH | 4 | 2770 | 82.5% | 586 | 17.5% | 3356 | 90.6% |
| EMB | 2 | 411 | 77.1% | 122 | 22.9% | 533 | 75.9% |
| EMB | 4 | 2361 | 93.5% | 163 | 6.5% | 2524 | 68.1% |
| PZA | 2 | 281 | 86.7% | 43 | 13.3% | 324 | 46.2% |
| PZA | 4 | 2019 | 95.6% | 92 | 4.4% | 2111 | 57.0% |
| STREP | 2 | 223 | 57.0% | 168 | 43.0% | 391 | 55.7% |
| STREP | 4 | 1499 | 84.7% | 271 | 15.3% | 1770 | 47.8% |
| CIP | 2 | 22 | 91.7% | 2 | 8.3% | 24 | 3.4% |
| CIP | 4 | 140 | 87.5% | 20 | 12.5% | 160 | 4.3% |
| MOX | 2 | 120 | 93.8% | 8 | 6.3% | 128 | 18.2% |
| MOX | 4 | 320 | 96.7% | 11 | 3.3% | 331 | 8.9% |
| OFL | 2 | 109 | 61.2% | 69 | 38.8% | 178 | 25.4% |
| OFL | 4 | 282 | 79.2% | 74 | 20.8% | 356 | 9.6% |
| KAN | 2 | 178 | 86.0% | 29 | 14.0% | 207 | 29.5% |
| KAN | 4 | 379 | 92.4% | 31 | 7.6% | 410 | 11.1% |
| AMK | 2 | 121 | 79.6% | 31 | 20.4% | 152 | 21.7% |
| AMK | 4 | 510 | 94.4% | 30 | 5.6% | 540 | 14.6% |
| CAP | 2 | 218 | 88.6% | 28 | 11.4% | 246 | 35.0% |
| CAP | 4 | 531 | 92.7% | 42 | 7.3% | 573 | 15.5% |
| ETH | 2 | 130 | 76.0% | 41 | 24.0% | 171 | 24.4% |
| ETH | 4 | 240 | 86.6% | 37 | 13.4% | 277 | 7.5% |
