# Supplementary material for: Genome-wide analysis of Mycobacterium tuberculosis polymorphisms reveals lineage-specific associations with drug resistance
Source: BMC Genomics. 2019 Mar 29;20:252. doi: 10.1186/s12864-019-5615-3 (PMC6440112; doi:10.1186/s12864-019-5615-3)
Supplement: Supplementary file 6 — Cross-resistance phenotype table, Cross-Resistance Table upper diagonal shows proportion of samples phenotyped for both vertical and horizontal phenotype, that test positive for vertical phenotype. Diagonal shows number of samples with each phenotype. Lower diagonal shows number of samples with phenotype for both horizontal and vertical phenotype. (PPTX 45 kb) [file 12864_2019_5615_MOESM6_ESM.pptx]

## Slide 1
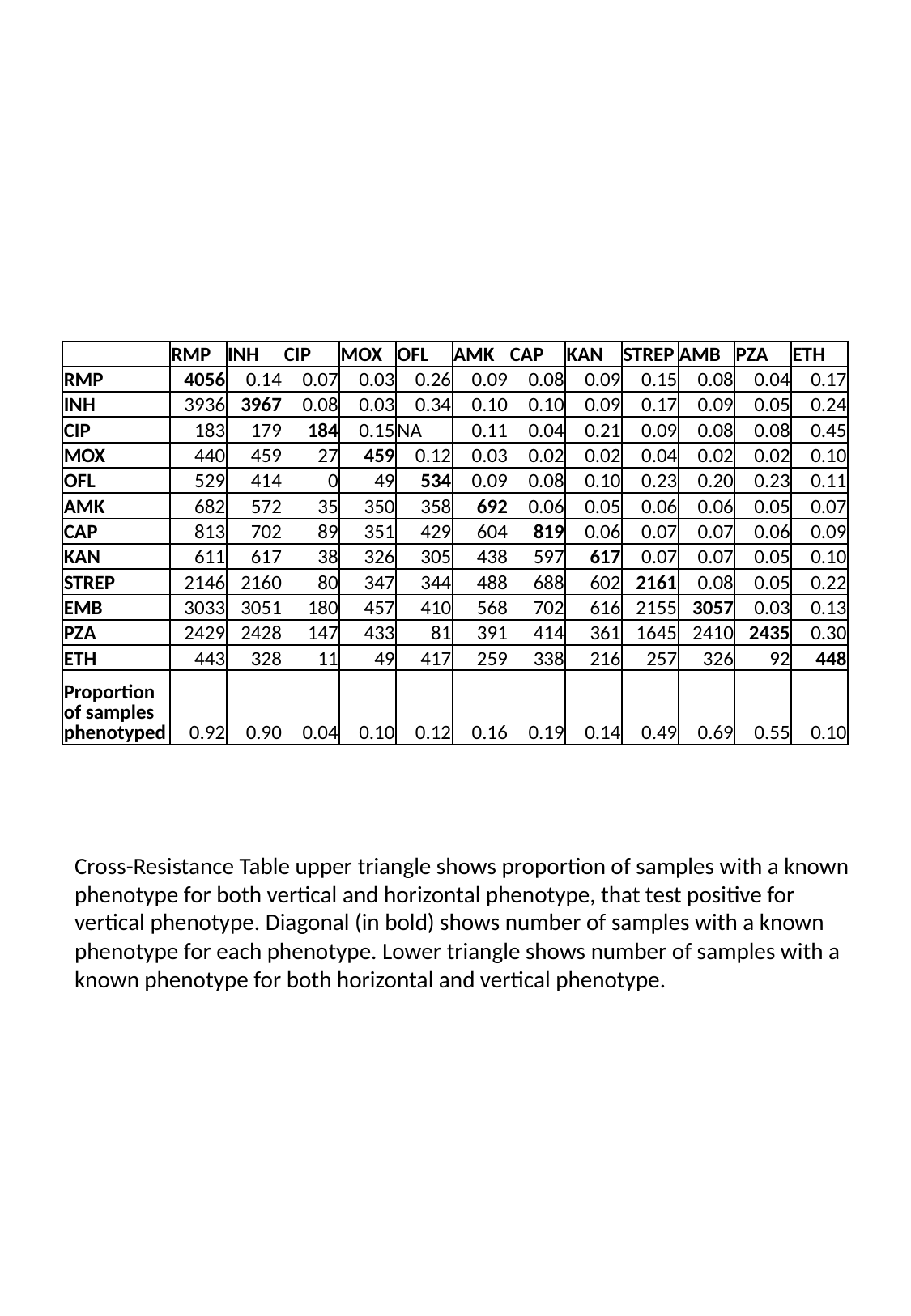

| | RMP | INH | CIP | MOX | OFL | AMK | CAP | KAN | STREP | AMB | PZA | ETH |
| --- | --- | --- | --- | --- | --- | --- | --- | --- | --- | --- | --- | --- |
| RMP | 4056 | 0.14 | 0.07 | 0.03 | 0.26 | 0.09 | 0.08 | 0.09 | 0.15 | 0.08 | 0.04 | 0.17 |
| INH | 3936 | 3967 | 0.08 | 0.03 | 0.34 | 0.10 | 0.10 | 0.09 | 0.17 | 0.09 | 0.05 | 0.24 |
| CIP | 183 | 179 | 184 | 0.15 | NA | 0.11 | 0.04 | 0.21 | 0.09 | 0.08 | 0.08 | 0.45 |
| MOX | 440 | 459 | 27 | 459 | 0.12 | 0.03 | 0.02 | 0.02 | 0.04 | 0.02 | 0.02 | 0.10 |
| OFL | 529 | 414 | 0 | 49 | 534 | 0.09 | 0.08 | 0.10 | 0.23 | 0.20 | 0.23 | 0.11 |
| AMK | 682 | 572 | 35 | 350 | 358 | 692 | 0.06 | 0.05 | 0.06 | 0.06 | 0.05 | 0.07 |
| CAP | 813 | 702 | 89 | 351 | 429 | 604 | 819 | 0.06 | 0.07 | 0.07 | 0.06 | 0.09 |
| KAN | 611 | 617 | 38 | 326 | 305 | 438 | 597 | 617 | 0.07 | 0.07 | 0.05 | 0.10 |
| STREP | 2146 | 2160 | 80 | 347 | 344 | 488 | 688 | 602 | 2161 | 0.08 | 0.05 | 0.22 |
| EMB | 3033 | 3051 | 180 | 457 | 410 | 568 | 702 | 616 | 2155 | 3057 | 0.03 | 0.13 |
| PZA | 2429 | 2428 | 147 | 433 | 81 | 391 | 414 | 361 | 1645 | 2410 | 2435 | 0.30 |
| ETH | 443 | 328 | 11 | 49 | 417 | 259 | 338 | 216 | 257 | 326 | 92 | 448 |
| Proportion of samples phenotyped | 0.92 | 0.90 | 0.04 | 0.10 | 0.12 | 0.16 | 0.19 | 0.14 | 0.49 | 0.69 | 0.55 | 0.10 |
Cross-Resistance Table upper triangle shows proportion of samples with a known phenotype for both vertical and horizontal phenotype, that test positive for vertical phenotype. Diagonal (in bold) shows number of samples with a known phenotype for each phenotype. Lower triangle shows number of samples with a known phenotype for both horizontal and vertical phenotype.
