# Supplementary material for: Genome-wide analysis of Mycobacterium tuberculosis polymorphisms reveals lineage-specific associations with drug resistance
Source: BMC Genomics. 2019 Mar 29;20:252. doi: 10.1186/s12864-019-5615-3 (PMC6440112; doi:10.1186/s12864-019-5615-3)
Supplement: Supplementary file 7 — Variant Position Table, Table detailing variants at all positions with at least one non-synonymous variant found to be significantly associated with a phenotype in any of the variant-based analyses. (PPTX 52 kb) [file 12864_2019_5615_MOESM7_ESM.pptx]

## Slide 1
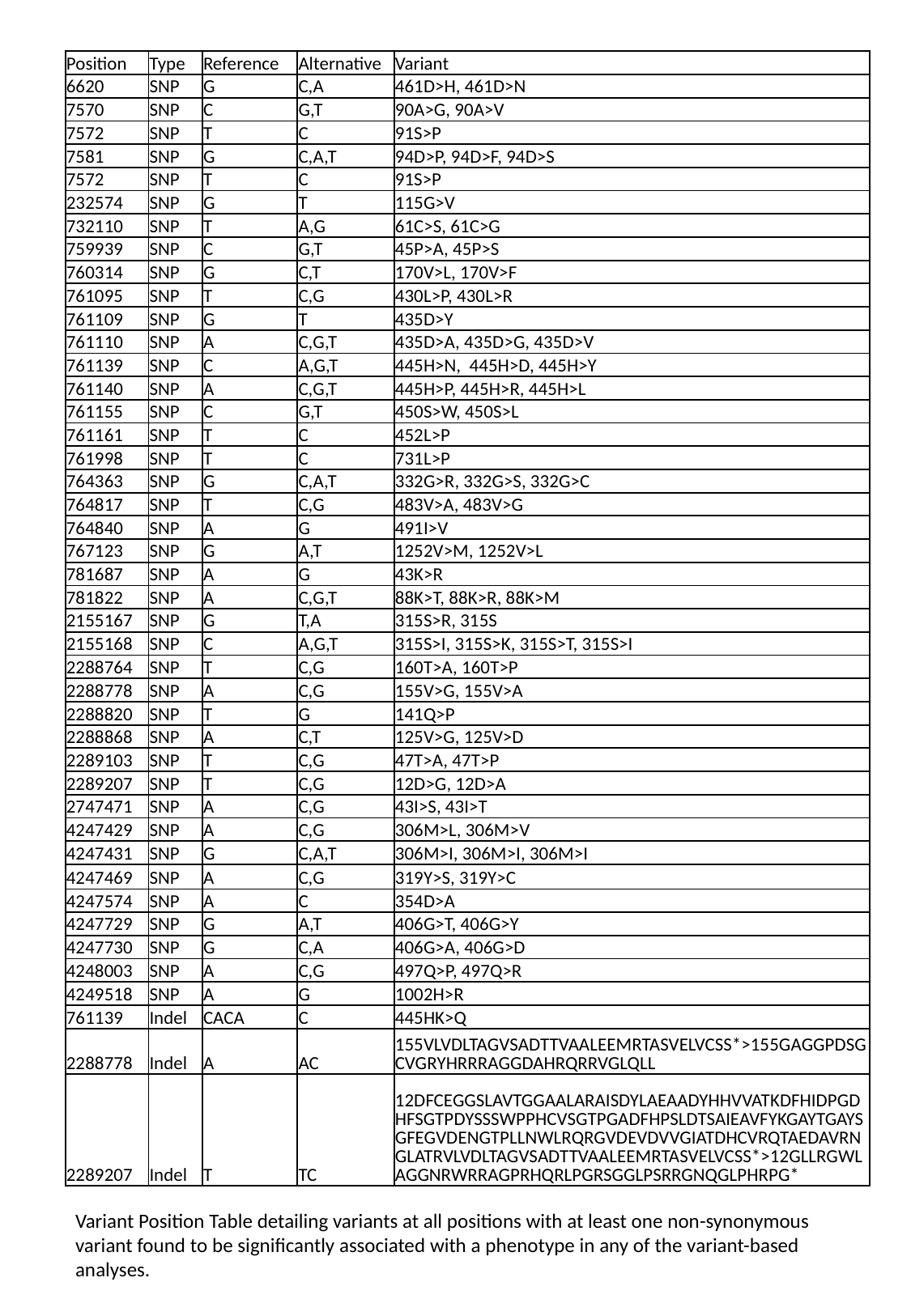

| Position | Type | Reference | Alternative | Variant |
| --- | --- | --- | --- | --- |
| 6620 | SNP | G | C,A | 461D>H, 461D>N |
| 7570 | SNP | C | G,T | 90A>G, 90A>V |
| 7572 | SNP | T | C | 91S>P |
| 7581 | SNP | G | C,A,T | 94D>P, 94D>F, 94D>S |
| 7572 | SNP | T | C | 91S>P |
| 232574 | SNP | G | T | 115G>V |
| 732110 | SNP | T | A,G | 61C>S, 61C>G |
| 759939 | SNP | C | G,T | 45P>A, 45P>S |
| 760314 | SNP | G | C,T | 170V>L, 170V>F |
| 761095 | SNP | T | C,G | 430L>P, 430L>R |
| 761109 | SNP | G | T | 435D>Y |
| 761110 | SNP | A | C,G,T | 435D>A, 435D>G, 435D>V |
| 761139 | SNP | C | A,G,T | 445H>N, 445H>D, 445H>Y |
| 761140 | SNP | A | C,G,T | 445H>P, 445H>R, 445H>L |
| 761155 | SNP | C | G,T | 450S>W, 450S>L |
| 761161 | SNP | T | C | 452L>P |
| 761998 | SNP | T | C | 731L>P |
| 764363 | SNP | G | C,A,T | 332G>R, 332G>S, 332G>C |
| 764817 | SNP | T | C,G | 483V>A, 483V>G |
| 764840 | SNP | A | G | 491I>V |
| 767123 | SNP | G | A,T | 1252V>M, 1252V>L |
| 781687 | SNP | A | G | 43K>R |
| 781822 | SNP | A | C,G,T | 88K>T, 88K>R, 88K>M |
| 2155167 | SNP | G | T,A | 315S>R, 315S |
| 2155168 | SNP | C | A,G,T | 315S>I, 315S>K, 315S>T, 315S>I |
| 2288764 | SNP | T | C,G | 160T>A, 160T>P |
| 2288778 | SNP | A | C,G | 155V>G, 155V>A |
| 2288820 | SNP | T | G | 141Q>P |
| 2288868 | SNP | A | C,T | 125V>G, 125V>D |
| 2289103 | SNP | T | C,G | 47T>A, 47T>P |
| 2289207 | SNP | T | C,G | 12D>G, 12D>A |
| 2747471 | SNP | A | C,G | 43I>S, 43I>T |
| 4247429 | SNP | A | C,G | 306M>L, 306M>V |
| 4247431 | SNP | G | C,A,T | 306M>I, 306M>I, 306M>I |
| 4247469 | SNP | A | C,G | 319Y>S, 319Y>C |
| 4247574 | SNP | A | C | 354D>A |
| 4247729 | SNP | G | A,T | 406G>T, 406G>Y |
| 4247730 | SNP | G | C,A | 406G>A, 406G>D |
| 4248003 | SNP | A | C,G | 497Q>P, 497Q>R |
| 4249518 | SNP | A | G | 1002H>R |
| 761139 | Indel | CACA | C | 445HK>Q |
| 2288778 | Indel | A | AC | 155VLVDLTAGVSADTTVAALEEMRTASVELVCSS\*>155GAGGPDSGCVGRYHRRRAGGDAHRQRRVGLQLL |
| 2289207 | Indel | T | TC | 12DFCEGGSLAVTGGAALARAISDYLAEAADYHHVVATKDFHIDPGDHFSGTPDYSSSWPPHCVSGTPGADFHPSLDTSAIEAVFYKGAYTGAYSGFEGVDENGTPLLNWLRQRGVDEVDVVGIATDHCVRQTAEDAVRNGLATRVLVDLTAGVSADTTVAALEEMRTASVELVCSS\*>12GLLRGWLAGGNRWRRAGPRHQRLPGRSGGLPSRRGNQGLPHRPG\* |
Variant Position Table detailing variants at all positions with at least one non-synonymous variant found to be significantly associated with a phenotype in any of the variant-based analyses.
