# Supplementary material for: Genome-wide analysis of Mycobacterium tuberculosis polymorphisms reveals lineage-specific associations with drug resistance
Source: BMC Genomics. 2019 Mar 29;20:252. doi: 10.1186/s12864-019-5615-3 (PMC6440112; doi:10.1186/s12864-019-5615-3)
Supplement: Supplementary file 8 — Locus Comparison Table, Locus comparison table showing which analyses and in which lineage each loci was identified. An ‘x’ indicates a locus which was not identified by the method of analysis in question. Loci without a known association with the phenotype are highlighted in bold. (PPTX 44 kb) [file 12864_2019_5615_MOESM8_ESM.pptx]

## Slide 1
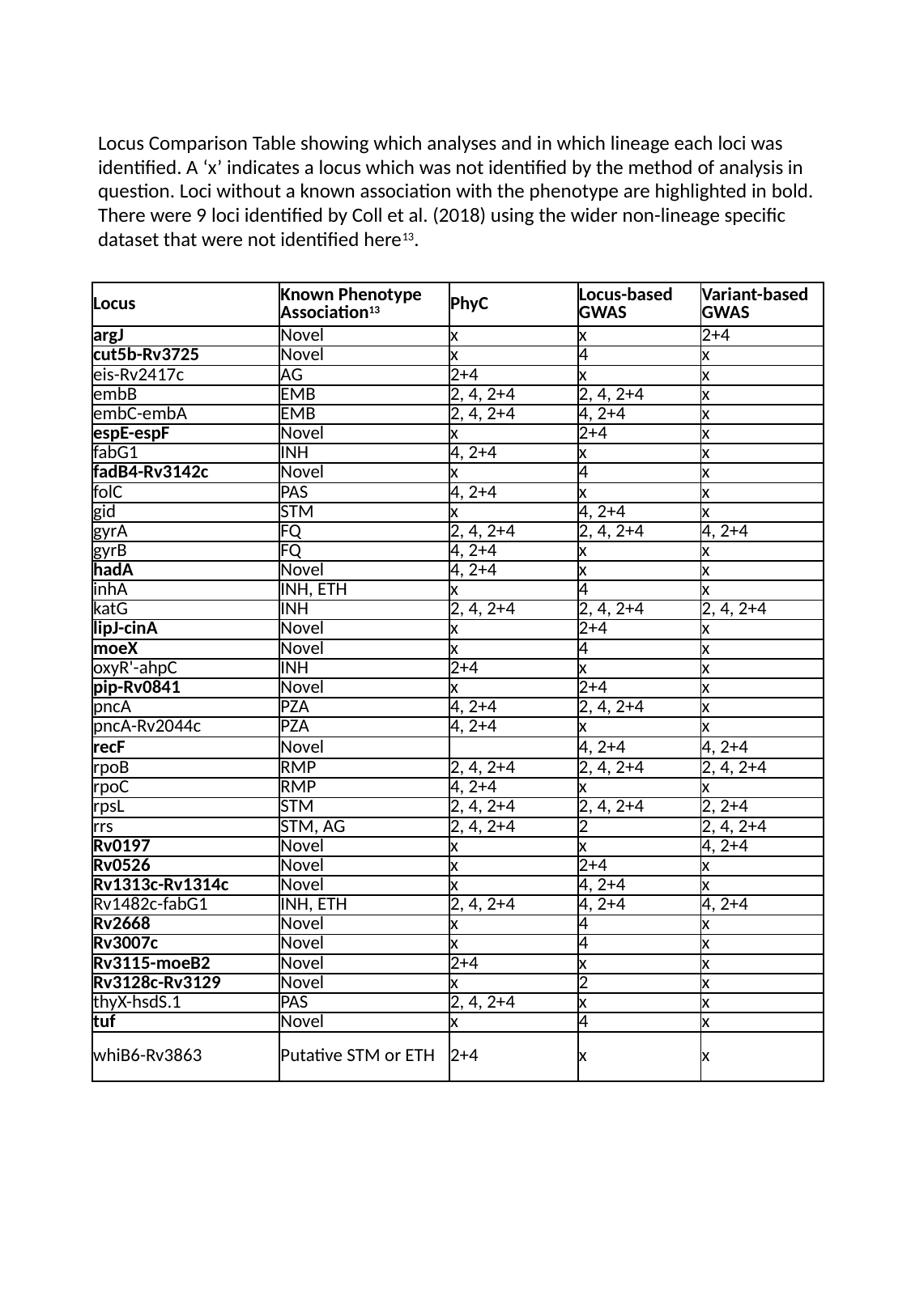

Locus Comparison Table showing which analyses and in which lineage each loci was identified. A ‘x’ indicates a locus which was not identified by the method of analysis in question. Loci without a known association with the phenotype are highlighted in bold. There were 9 loci identified by Coll et al. (2018) using the wider non-lineage specific dataset that were not identified here13.
| Locus | Known Phenotype Association13 | PhyC | Locus-based GWAS | Variant-based GWAS |
| --- | --- | --- | --- | --- |
| argJ | Novel | x | x | 2+4 |
| cut5b-Rv3725 | Novel | x | 4 | x |
| eis-Rv2417c | AG | 2+4 | x | x |
| embB | EMB | 2, 4, 2+4 | 2, 4, 2+4 | x |
| embC-embA | EMB | 2, 4, 2+4 | 4, 2+4 | x |
| espE-espF | Novel | x | 2+4 | x |
| fabG1 | INH | 4, 2+4 | x | x |
| fadB4-Rv3142c | Novel | x | 4 | x |
| folC | PAS | 4, 2+4 | x | x |
| gid | STM | x | 4, 2+4 | x |
| gyrA | FQ | 2, 4, 2+4 | 2, 4, 2+4 | 4, 2+4 |
| gyrB | FQ | 4, 2+4 | x | x |
| hadA | Novel | 4, 2+4 | x | x |
| inhA | INH, ETH | x | 4 | x |
| katG | INH | 2, 4, 2+4 | 2, 4, 2+4 | 2, 4, 2+4 |
| lipJ-cinA | Novel | x | 2+4 | x |
| moeX | Novel | x | 4 | x |
| oxyR'-ahpC | INH | 2+4 | x | x |
| pip-Rv0841 | Novel | x | 2+4 | x |
| pncA | PZA | 4, 2+4 | 2, 4, 2+4 | x |
| pncA-Rv2044c | PZA | 4, 2+4 | x | x |
| recF | Novel | | 4, 2+4 | 4, 2+4 |
| rpoB | RMP | 2, 4, 2+4 | 2, 4, 2+4 | 2, 4, 2+4 |
| rpoC | RMP | 4, 2+4 | x | x |
| rpsL | STM | 2, 4, 2+4 | 2, 4, 2+4 | 2, 2+4 |
| rrs | STM, AG | 2, 4, 2+4 | 2 | 2, 4, 2+4 |
| Rv0197 | Novel | x | x | 4, 2+4 |
| Rv0526 | Novel | x | 2+4 | x |
| Rv1313c-Rv1314c | Novel | x | 4, 2+4 | x |
| Rv1482c-fabG1 | INH, ETH | 2, 4, 2+4 | 4, 2+4 | 4, 2+4 |
| Rv2668 | Novel | x | 4 | x |
| Rv3007c | Novel | x | 4 | x |
| Rv3115-moeB2 | Novel | 2+4 | x | x |
| Rv3128c-Rv3129 | Novel | x | 2 | x |
| thyX-hsdS.1 | PAS | 2, 4, 2+4 | x | x |
| tuf | Novel | x | 4 | x |
| whiB6-Rv3863 | Putative STM or ETH | 2+4 | x | x |
