# Supplementary material for: Genome-wide analysis of Mycobacterium tuberculosis polymorphisms reveals lineage-specific associations with drug resistance
Source: BMC Genomics. 2019 Mar 29;20:252. doi: 10.1186/s12864-019-5615-3 (PMC6440112; doi:10.1186/s12864-019-5615-3)
Supplement: Supplementary file 9 — Study frequency table, Study frequency table, showing numbers and percentage of strains from each study by lineage. (PPTX 40 kb) [file 12864_2019_5615_MOESM9_ESM.pptx]

## Slide 1
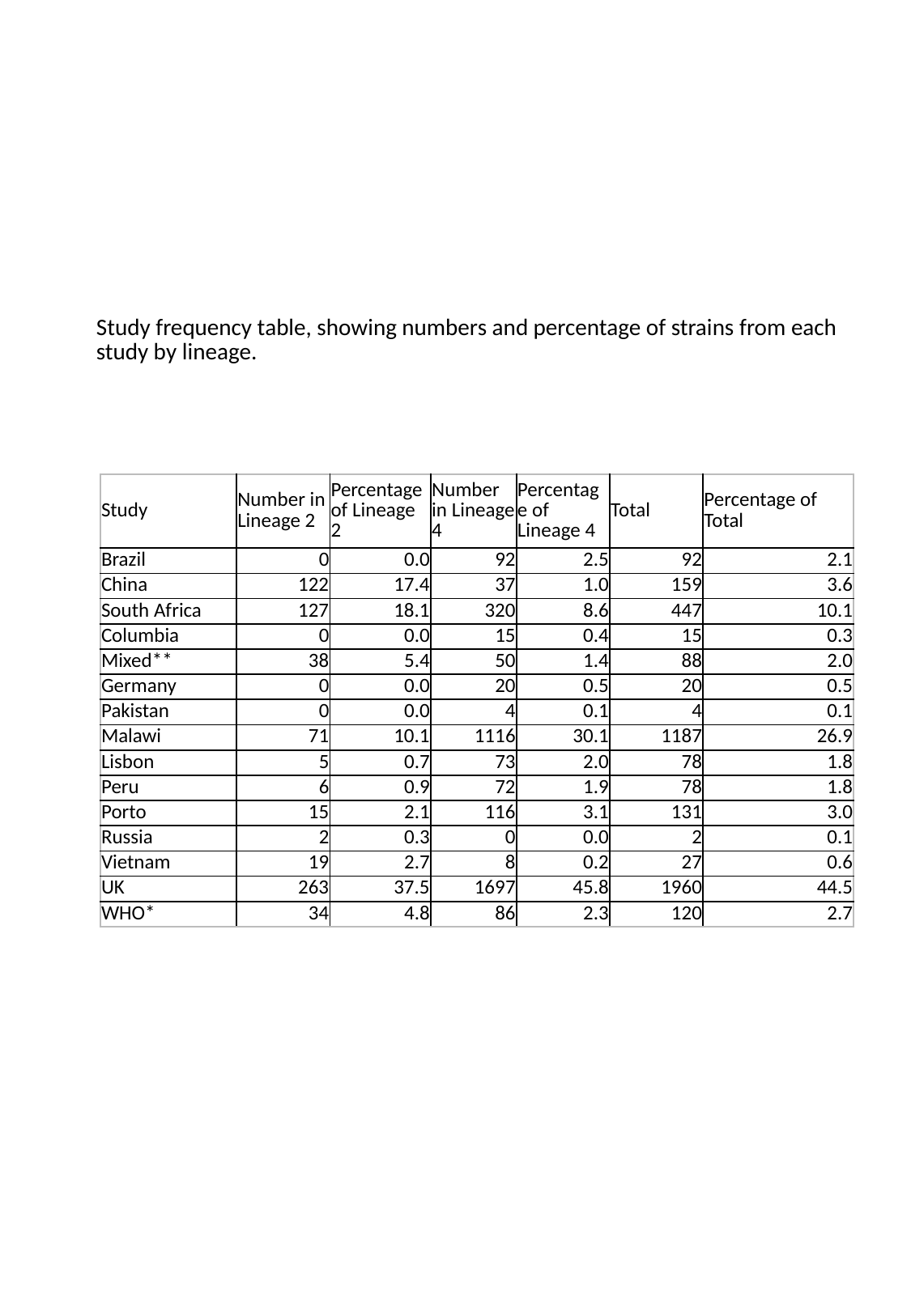

Study frequency table, showing numbers and percentage of strains from each study by lineage.
| Study | Number in Lineage 2 | Percentage of Lineage 2 | Number in Lineage 4 | Percentage of Lineage 4 | Total | Percentage of Total |
| --- | --- | --- | --- | --- | --- | --- |
| Brazil | 0 | 0.0 | 92 | 2.5 | 92 | 2.1 |
| China | 122 | 17.4 | 37 | 1.0 | 159 | 3.6 |
| South Africa | 127 | 18.1 | 320 | 8.6 | 447 | 10.1 |
| Columbia | 0 | 0.0 | 15 | 0.4 | 15 | 0.3 |
| Mixed\*\* | 38 | 5.4 | 50 | 1.4 | 88 | 2.0 |
| Germany | 0 | 0.0 | 20 | 0.5 | 20 | 0.5 |
| Pakistan | 0 | 0.0 | 4 | 0.1 | 4 | 0.1 |
| Malawi | 71 | 10.1 | 1116 | 30.1 | 1187 | 26.9 |
| Lisbon | 5 | 0.7 | 73 | 2.0 | 78 | 1.8 |
| Peru | 6 | 0.9 | 72 | 1.9 | 78 | 1.8 |
| Porto | 15 | 2.1 | 116 | 3.1 | 131 | 3.0 |
| Russia | 2 | 0.3 | 0 | 0.0 | 2 | 0.1 |
| Vietnam | 19 | 2.7 | 8 | 0.2 | 27 | 0.6 |
| UK | 263 | 37.5 | 1697 | 45.8 | 1960 | 44.5 |
| WHO\* | 34 | 4.8 | 86 | 2.3 | 120 | 2.7 |
