# Supplementary material for: Genome-wide analysis of Mycobacterium tuberculosis polymorphisms reveals lineage-specific associations with drug resistance
Source: BMC Genomics. 2019 Mar 29;20:252. doi: 10.1186/s12864-019-5615-3 (PMC6440112; doi:10.1186/s12864-019-5615-3)
Supplement: Supplementary file 10 — Sublineage frequency table, Numbers and percentage by lineage assigned to each sublineage. (PPTX 36 kb) [file 12864_2019_5615_MOESM10_ESM.pptx]

## Slide 1
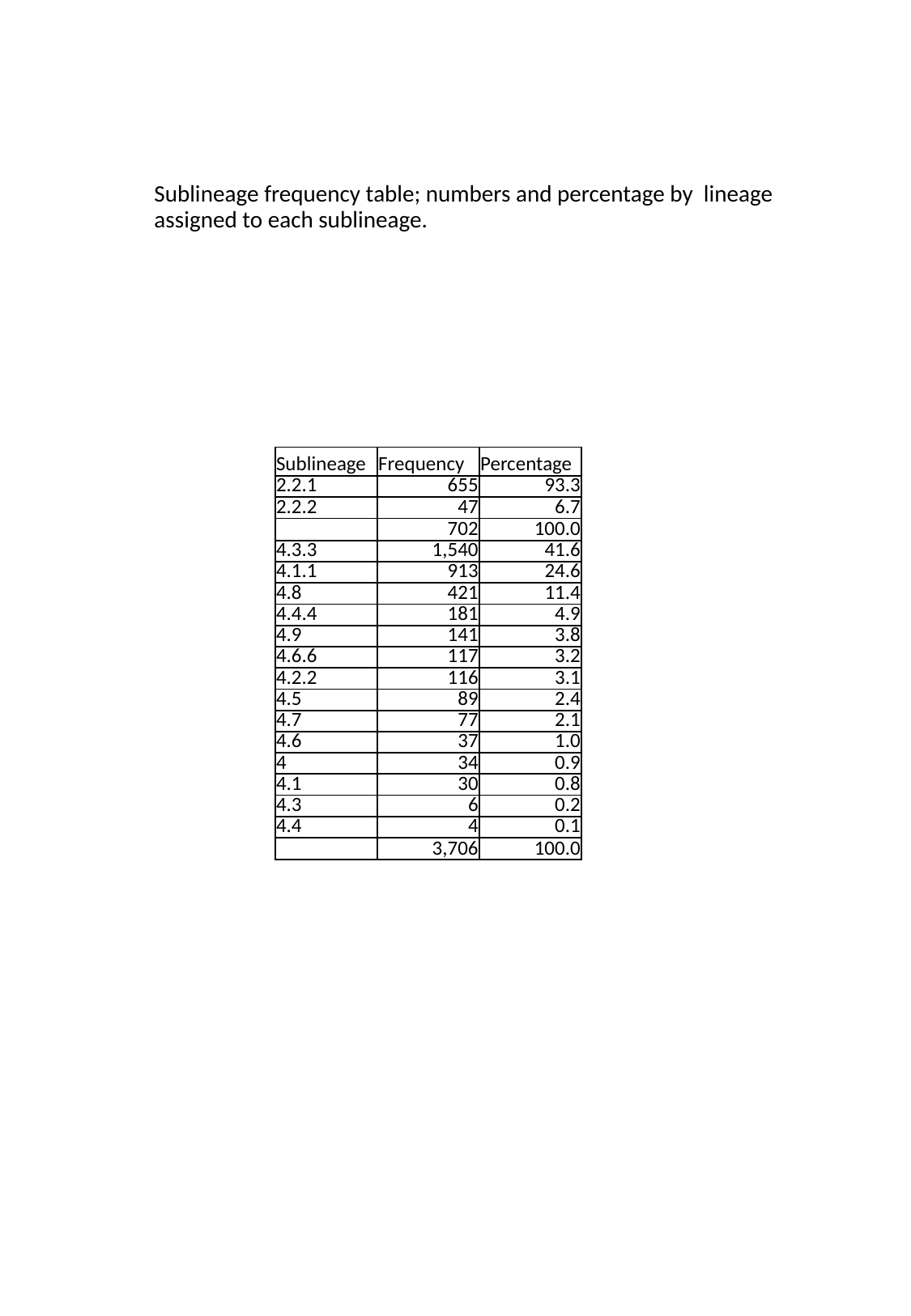

Sublineage frequency table; numbers and percentage by lineage assigned to each sublineage.
| Sublineage | Frequency | Percentage |
| --- | --- | --- |
| 2.2.1 | 655 | 93.3 |
| 2.2.2 | 47 | 6.7 |
| | 702 | 100.0 |
| 4.3.3 | 1,540 | 41.6 |
| 4.1.1 | 913 | 24.6 |
| 4.8 | 421 | 11.4 |
| 4.4.4 | 181 | 4.9 |
| 4.9 | 141 | 3.8 |
| 4.6.6 | 117 | 3.2 |
| 4.2.2 | 116 | 3.1 |
| 4.5 | 89 | 2.4 |
| 4.7 | 77 | 2.1 |
| 4.6 | 37 | 1.0 |
| 4 | 34 | 0.9 |
| 4.1 | 30 | 0.8 |
| 4.3 | 6 | 0.2 |
| 4.4 | 4 | 0.1 |
| | 3,706 | 100.0 |
